# Polyphasic Characterization of the Biocontrol Potential of a Novel Strain of *Trichoderma atroviride* Isolated from Central Mexico

**DOI:** 10.3390/jof10110758

**Published:** 2024-11-01

**Authors:** Karla Ivonne González-Martínez, Ma. Soledad Vázquez-Garcidueñas, Alfredo Herrera-Estrella, Sylvia P. Fernández-Pavía, Rafael Salgado-Garciglia, John Larsen, Salvador Ochoa-Ascencio, Gerardo Rodríguez-Alvarado, Gerardo Vázquez-Marrufo

**Affiliations:** 1 Multidisciplinary Center for Biotechnology Studies, Faculty of Veterinary Medicine and Zootechnics, Universidad Michoacana de San Nicolás de Hidalgo, Km 9.5 Carretera Morelia-Zinapécuaro, Col. La Palma, Tarímbaro CP 58893, Michoacán, Mexico; karla.ivonne.gonzalez@umich.mx; 2 Division of Graduate Studies, Faculty of Medical and Biological Sciences “Dr. Ignacio Chávez”, Universidad Michoacana de San Nicolás de Hidalgo, Ave. Rafael Carrillo esq. Dr. Salvador González Herrejón, Col. Cuauhtémoc, Morelia CP 58020, Michoacán, Mexico; soledad.vazquez@umich.mx; 3 Unidad de Genómica Avanzada-Langebio, Centro de Investigación y de Estudios Avanzados del IPN, Libramiento Norte Carretera Irapuato-León km 9.6, Irapuato CP 36824, Guanajuato, Mexico; alfredo.herrera@cinvestav.mx; 4 Institute of Research in Agricultural and Forestry Sciences, Universidad Michoacana de San Nicolás de Hidalgo, Km 9.5 Carretera Morelia-Zinapécuaro, Col. La Palma, Tarímbaro CP 58893, Michoacán, Mexico; patricia.pavia@umich.mx (S.P.F.-P.); gerardo.rodriguez@umich.mx (G.R.-A.); 5 Chemical Biological Research Institute, Universidad Michoacana de San Nicolás de Hidalgo, Av. Universidad s/n, Ciudad Universitaria, Morelia CP 58069, Michoacán, Mexico; rafael.salgado@umich.mx; 6 Ecosystem and Sustainability Research Institute, Universidad Nacional Autónoma de México, Antigua Carretera a Pátzcuaro No.8701, Col. Ex Hacienda de San José de la Huerta, Morelia CP 58190, Michoacán, Mexico; jlarsen@cieco.unam.mx; 7 Faculty of Agrobiology, Universidad Michoacana de San Nicolás de Hidalgo, Paseo Lázaro Cárdenas 2290, Emiliano Zapata, Melchor Ocampo, Uruapan CP 60170, Michoacán, Mexico; salvador.ochoa@umich.mx

**Keywords:** antagonism, *Botrytis cinerea*, detached leaf assay, mycoparasitism, hydrolytic enzymes, transcriptional activation

## Abstract

This work describes the characterization of *Trichoderma atroviride* strain CMU-08, isolated from Michoacán, Mexico. CMU-08 demonstrated robust growth and conidiation across a temperature range from 16 to 32 °C and a pH range from 4 to 9 on potato dextrose agar (PDA) and malt extract agar (MEA) media. The strain is an efficient antagonist of six species of phytopathogenic fungi and oomycetes in PDA, MEA, and Vogel minimal medium (VMM). Antagonist mechanisms of CMU-08 included direct mycoparasitism observed in dual-culture assays, as well as antibiosis attributed to growth inhibition via both volatile and non-volatile metabolites, with the effectiveness varying depending on the test phytopathogen and culture medium. Extracellular filtrates (ECFs) recovered from liquid cultures of CMU-08 under basal and induced conditions using *Botrytis cinerea* cell walls significantly inhibited their growth at a concentration of 750 µg/mL. Moreover, in detached tomato leaf assays, these ECFs reduced foliar damage caused by *B. cinerea* by 24–34%. The volatile organic compounds (VOCs) produced by CMU-08 also exhibited substantial efficacy, reducing foliar damage by up to 50% in similar tests. Despite showing no basal extracellular chitinase enzymatic activity, CMU-08 demonstrated significant induction of this activity in cultures supplemented with *B. cinerea* and *Fusarium* sp. cell walls. Four genes encoding extracellular chitinases (*chit33*, *chit36*, *ech42*, and locus 217415) showed different dynamics of transcriptional regulation during the dual-culture confrontation of strain CMU-08 with *B. cinerea* and *Fusarium* sp., varying according to the phytopathogen and the interaction stage. The CMU-08 strain shows physiological versatility and employs a variety of antagonist mechanisms toward different species of phytopathogenic microorganisms, making it a good candidate for developing a biocontrol product for field application.

## 1. Introduction

The agricultural sector represents vital human activity, generating 11 billion tons of food annually worldwide. It also provides a quarter of jobs globally, and in low-income countries it represents around 60% of the workforce [1]. Humanity depends on agricultural production to meet its current and future basic food requirements [2], which must face the challenge of increasing production while minimizing the environmental impact caused by the sector’s activities [3,4]. One of the main factors affecting global agricultural production is infectious diseases of plants, which cause significant losses [5,6]. These diseases are caused by different microbial agents, including viruses, bacteria, and fungi, with the latter being the predominant agent affecting agricultural production [7]. To date, these phytopathogens have been managed using agrochemicals [8,9]. However, because of their chemical nature and lack of specificity, agrochemicals adversely affect human and animal health. They also disrupt ecosystem processes due to toxicity and bioaccumulation [7,10]. Furthermore, some species of fungi and oomycetes have developed resistance to certain pesticides used for their control [8,11,12,13,14]. As a result, alternative approaches to agrochemicals have been explored, with biocontrol emerging as a major component of integrated management strategies [15,16,17].

Several species within the *Trichoderma* genus have been recognized as efficient antagonists against phytopathogenic microorganisms, particularly fungi and oomycetes [18,19]. While biocontrol products containing *Trichoderma* species have been successfully commercialized [20], the ongoing task of isolating and characterizing new strains of this genus persists. This is primarily due to the extensive diversity of phytopathogen species that need to be targeted, their widespread prevalence globally [21,22], and the intraspecific variations observed among strains across different crop types or geographical regions [23,24,25,26,27,28,29]. Additionally, shifts in local climatic conditions resulting from global climate change, necessitate the search for specially adapted strains suitable for biocontrol in crops within specific geographic regions [30,31,32,33].

The effectiveness of *Trichoderma* spp. as a biocontrol agent stems from their diverse mechanisms to combat and inhibit the growth of phytopathogenic microorganisms [7,34,35]. Within the *Trichoderma* genus, the reference strains IMI206040 and P1 of *Trichoderma atroviride* have been used as models to study the biochemical, physiological, and molecular genetic bases of the various mechanisms associated with biocontrol capacity in the field. In this context, antagonism in dual cultures against different phytopathogens, the activity of extracellular hydrolytic enzymes involved in mycoparasitism, and the production of secondary metabolites with antifungal activity—both volatile organic compounds (VOCs) and soluble—have been evaluated in these strains under different culture conditions [36,37,38,39,40,41,42,43,44]. However, the isolation and in vitro characterization of *T. atroviride* strains from different geographic regions have documented the significant genetic diversity and intraspecific physiological plasticity of this species, even among genomically identical strains [45,46,47,48,49,50]. This analysis of wild strains from around the world highlights the importance of continuing to isolate and characterize new *T. atroviride* strains in vitro, particularly from regions with high biological diversity that are still scarcely explored, such as Mexico. Therefore, assessing new *Trichoderma* strains with biocontrol potential in agricultural settings should involve conducting tests to demonstrate their physiological and biochemical capabilities related to various antagonistic mechanisms [51,52]. This approach, which evaluates *Trichoderma* spp. strains with biocontrol potential using a combination of methods, is referred to as polyphasic characterization [53,54].

This study aimed to conduct a comprehensive polyphasic characterization of the CMU-08 strain of *Trichoderma atroviride*, previously isolated and identified by our research group [55]. This work integrates various elements of analysis that have not been combined in previous studies. Physiological, metabolic, biochemical, and molecular-genetic tests assessed the strain’s application in agricultural settings. This study represents one of the most detailed investigations into the multifaceted characterization of a *T. atroviride* strain isolated in Mexico, highlighting its potential for biocontrol applications. Furthermore, the results enhance our understanding of the intraspecific variability of *T. atroviride* on a global scale, contributing significantly to the ecological and evolutionary insights into this biotechnologically important species.

## 2. Materials and Methods

### 2.1. Analyzed Strains

*Trichoderma atroviride* strain CMU-08 was isolated from the “José Ma. Morelos” National Park, in the municipality of Charo, Michoacán [55], and is maintained in the Michoacan University Culture Collection (CMU), housed at the Microorganism Conservation and Biotechnology Laboratory, Multidisciplinary Center for Biotechnology Studies, Facultad de Medicina Veterinaria y Zootecnia, Universidad Michoacana de San Nicolás de Hidalgo (Michoacán, Mexico). The identification of this strain as *T. atroviride* was carried out through phylogenetic analysis using the ITS region (Genbank accession number: KR607462.1) and the *tef1* (Genbank accession number: KT287049.1) gene [55] and has been subsequently corroborated by genome assembly (manuscript in preparation). Phytopathogenic strains used for antagonism and inhibition assays included six species of fungi and one oomycete species from the genus *Phytophthora*, isolated from various crops in Michoacán, Mexico (Table 1).

### 2.2. Inoculum Generation

Inocula for all growth kinetics and enzymatic activity assays were obtained from actively growing vegetative mycelium colonies. The strain was cultured on potato dextrose agar medium (PDA, BD Difco, Sparks, MD, USA) at 28 °C until the colonies reached the linear growth phase. Inocula were harvested from the colony edge using a 6 mm diameter punch. Similar methods were used to prepare inoculum for all tested phytopathogens in solid medium assays, including *Botrytis cinerea* for inhibition assays.

### 2.3. Growth Kinetics

#### 2.3.1. Growth Kinetics in Solid Medium

Inocula were centrally placed on 95 mm diameter Petri dishes containing PDA or malt extract agar (MEA, BD Difco, USA) and incubated in darkness under varying temperature and pH conditions. The acidity (pH 4.0) of the media was adjusted with HCl 0.1N, whereas the media with neutral (7.0) or basic (9.0) pH were adjusted with NaOH 1N. The diameter of mycelial colonies was measured every 24 h for ten days until the strain’s mycelium entirely covered the culture medium’s surface. Conidial development initiation under each culture condition was visually recorded. All experiments were conducted in triplicate.

#### 2.3.2. Growth Kinetics in Liquid Medium

Growth kinetics were performed in Vogel’s minimal medium (VMM) [55], serving as the basal culture (BC) conditions, with additional supplementation of inactivated *B. cinerea* mycelium at 1% (*w*/*v*), serving as the induced culture (IC) conditions. *Botrytis cinerea* mycelium, obtained from potato dextrose broth medium (PDB, BD Difco, USA), was inactivated by autoclaving and subsequently lyophilized for use as a supplement in IC. Cultures were incubated in 125 mL Erlenmeyer flasks containing 25 mL of medium, inoculated with three CMU-08 strain inocula as described earlier, and incubated at 28 °C with 120 rpm of agitation. Biomass (mycelium) was recovered by filtration, and the dry weight was determined over a 10-day incubation period. All experiments were performed in triplicate.

### 2.4. Confrontation Assays in Dual Culture

The method described by Cherif et al. [56] was employed to evaluate the antagonistic activity of the CMU-08 strain against the test phytopathogens. Assays were conducted on PDA, MEA, and VMM media. Petri dishes were inoculated with the CMU-08 strain and various phytopathogens and incubated at 28 °C in darkness. Growth controls of each phytopathogen were used to determine the extent of antagonism [57], as follows: 1 = complete overgrowth by *T. atroviride*; 2 = two-thirds overgrowth; and 3 = equal growth without dominance.

### 2.5. Assays of the Growth Inhibition of Phytopathogens

#### 2.5.1. Inhibition by Non-Volatile Metabolites

The method of Dennis et al. [58] was followed, placing sterilized cellophane membranes (90 mm diameter) on the culture medium. CMU-08 was inoculated at the center of Petri dishes, and once the mycelium covered three-quarters of the medium’s surface, the cellophane paper covered with the mycelium was removed. In independent experiments, each phytopathogenic microorganism was inoculated and incubated at 28 °C. Radial growth diameters were measured until the control cultures were fully covered. The percentage of inhibition of mycelial growth was calculated using the formula % inhibition = [(D1 − D2)/D1 × 100] [59], where D1 = colony diameter of phytopathogenic microorganism in medium free of inhibitors, and D2 = diameter of the same microorganism growing in plates with medium in which *T. atroviride* previously grew on cellophane paper. The type of inhibition was classified according to its level of activity, where class 1 = *T. atroviride* inhibits 67 to 100% of the growth of the phytopathogen, class 2 = *T. atroviride* inhibits 34 to 66% of the growth of the phytopathogen, and class 3 = *T. atroviride* inhibits 1 to 33% of the growth of the phytopathogen.

#### 2.5.2. Inhibition by Volatile Metabolites (VOCs)

This assay followed the method outlined by Nutter et al. [60]. Petri dishes containing VMM were individually inoculated with mycelium from each phytopathogen species and the CMU-08 strain. The lid of the Petri dish containing the *T. atroviride* strain was replaced with the bottom of the Petri dishes inoculated with the phytopathogens, serving as the cover for the plate containing *T. atroviride*. The junction between the plates was sealed with parafilm and incubated at 28 °C. The test concluded when the pathogen covered the control plate, excluding *T. atroviride* from the base. Inhibition percentages were calculated using the formula described earlier [59].

### 2.6. Scanning Electron Microscopy of Confrontation Assays

Confrontation assays were conducted using the CMU-08 strain against two phytopathogens previously mentioned. A 6 mm agar disc was extracted from the contact zone between the two strains and dried at 60 °C for 48 h. Interaction structures were observed using a JEOL JSPM-5200 microscope (Tokyo, Japan) operating at 20.0 kV.

### 2.7. In Vitro Inhibition Assays of Botrytis cinerea with the Extracellular Filtrate of the Liquid Culture of T. atroviride

Flasks of 500 mL containing 125 mL of basal (BC) and induced (IC) VMM were inoculated with 36 inocula obtained as previously described and incubated at 28 °C with shaking at 120 rpm for six days. After incubation, the mycelium was removed by filtration, and the resulting filtrate was sterilized using 0.45 µm pore size membranes (Merck, Kenilworth, NJ, USA). This filtrate was then used to supplement solid VMM at concentrations of 5 and 10% (*v*/*v*). The supplemented VMM was poured into 95 mm Petri dishes and inoculated at the center with a *B. cinerea* inoculum. The experiment included the following two control conditions: one with VMM plates without any added filtrate and another with the filtered medium that had not been previously inoculated with *T. atroviride*. The experiment concluded when the mycelium of the phytopathogen in the control plates had completely covered the surface of the control medium. The percentage of inhibition was determined using the formula described previously [59].

### 2.8. Inhibition of Phytopathogens Using Concentrates of the Extracellular Filtrate from T. atroviride Liquid Culture

The extracellular medium from *T. atroviride* cultures under basal (BC) and induced (IC) conditions was obtained by filtration and then concentrated to half its original volume using a rotary evaporator at 70 °C. The concentrated filtrate was subsequently sterilized by filtration, as previously described. The inhibitory effect of this concentrate on *B. cinerea* was evaluated on 96-well microplates, with a final volume of 100 µL per well of the concentrated filtrate. Growth inhibition was assessed by measuring the absorbance at 750 nm using a MicroStation instrument (Biolog^®^, Hayward, CA, USA) on the fourth day of incubation, and data were analyzed using the Microlog3™ Release 4.20 program.

*B. cinerea* was cultured on 95 mm Petri dishes with MEA medium and incubated at 28 °C for the phytopathogen. Afterward, the mycelium was collected and transferred to a borosilicate test tube containing 16 mL of IF-FF solution (0.25% Phytagel [Sigma, St. Louis, MO, USA], 0.03% Tween 40 [Sigma, USA], and H_2_O_dde_). The suspension was adjusted to achieve a transmittance of 75 ± 2 at a wavelength of 590 nm.

For the inhibition tests, 90 and 85 µL of the *B. cinerea* suspension were mixed with 10 µL and 15 µL of *T. atroviride* culture concentrate to obtain final 10 and 15% (*v*/*v*) concentrations, respectively. Two control treatments were included; the first consisted of 100 µL of *B. cinerea* inoculum in the well to monitor its growth, while the second included 90 µL and 85 µL of IF-FF solution plus 10 µL and 15 µL of BC and IC culture media concentrates, respectively, without the *T. atroviride* strain. The microplates were incubated at 28 °C for seven days.

The percentage of inhibition was calculated using the following formula:

Percent Inhibition = [(D1 − D2)/D1 × 100]

where D1 is the optical density of *B. cinerea* growing in the inhibitor-free medium, and D2 is the optical density of *B. cinerea* growing in the concentrated extracellular medium of *T. atroviride*.

### 2.9. Inhibition of Phytopathogens with Lyophilized Extracellular Filtrate from Liquid Culture of T. atroviride

Extracellular filtrates from each *T. atroviride* culture condition were lyophilized and re-suspended in IF-FF at the desired test concentrations. The *B. cinerea* strain was inoculated on MEA to obtain the inoculum in IF-FF. Microplate wells were filled with the phytopathogen inoculum and the lyophilized suspension, obtaining a final volume of 100 µL per well. Two control wells were used; the first contained 100 µL of the test strain inoculum in a microwell, and the second contained culture medium under BC and IC conditions that were not inoculated in two independent microwells. The prepared microplates were then incubated at 28 °C. Phytopathogen growth and the inhibition percentage were determined using the methodology described in previous tests. Additionally, the LC_50_ was calculated by performing a linear regression between the percentage of inhibition and the concentrations of the lyophilized products (mg/L).

### 2.10. Botrytis Cinerea Growth Inhibition Tests on Leaf Tissue

#### 2.10.1. Inhibition Assays by Soluble Metabolites

These assays utilized tomato leaves (*Solanum lycopersicum* var. Río Grande) of uniform size and development stage. Extracellular filtrates from *T. atroviride* were sterilized by filtration through 0.45 µm pore diameter membranes (Millipore, Billerica, MA, USA) and lyophilized. In independent trials, 50 µL of the lyophilized material resuspended in distilled water from each culture condition was applied to tomato leaves at 250 and 500 µg/mL concentrations. A 6 mm cylindrical inoculum of *B. cinerea* was placed on the leaves.

For controls, lyophilized and resuspended control media from each incubation condition without *T. atroviride* inoculation were applied in one instance, and no filtrate was applied in the second control. Petri dishes prepared under each condition were sealed and incubated at 28 °C for four days. At the end of the incubation period, the severity of damage caused by the phytopathogen on each leaf was assessed by calculating the percentage severity using the following formula: % severity = [(affected area/total area) × 100] [60]. The affected and total areas of the leaves were determined using the ImageJ software (https://imagej.net/ij/, National Institutes of Health, Bethesda, MD, USA).

#### 2.10.2. Inhibition Assays by VOCs

This assay followed the humid chamber method described by Sarven et al. [61]. Tomato leaves inoculated with *B. cinerea* were placed inside a 95 mm diameter Petri dish on a filter paper disk saturated with distilled water. The *T. atroviride* strain was inoculated in VMM medium in a Petri dish placed within the chamber to emit volatiles. A control plate containing a non-inoculated VMM medium was also included.

The humid chamber was sealed and incubated at room temperature for seven days. At the end of the incubation, the severity of damage caused by *B. cinerea* on each leaf was assessed by calculating the percentage severity using the previously described formula [60].

### 2.11. Quantitative Determination of the Enzymatic Activity of Chitinase

The enzymatic activity of cultures under BC and IC conditions was assessed by inducing the IC condition with cell walls from phytopathogens *B. cinerea* and *Fusarium* sp. at 0.5% (*w*/*v*). The methodology for constructing the enzymatic activity kinetics followed Qualhato et al. [62].

Flasks with 50 mL of medium were inoculated with six 6 mm diameter cylindrical inocula of *T. atroviride* and incubated at 28 °C, with shaking at 120 rpm. Aliquots of 1 mL of extracellular medium were collected every six hours for 48 h.

Chitinase activity was determined using 0.5% (*w*/*v*) colloidal chitin (Sigma, USA) in 50 mM acetate buffer, pH 5.5, as a substrate. The reaction mixture consisted of 50 μL of enzyme solution (extracellular medium) and 150 μL of colloidal chitin, which was incubated at 35 °C for 120 min. The reaction was stopped by adding 1 mL of 3,5-dinitrosalicylic acid (DNS) and incubating at 100 °C. The increase in absorbance at 540 nm (ε_540_ = 0.115293 mM^−1^ cm^−1^) due to the conversion of DNS to 3-amino-5-nitrosalicylic acid indicated an increase in reducing sugars. Enzyme activities are expressed in units (U), where one unit of enzyme activity liberates 1 μmoL of reducing sugars per minute.

### 2.12. Chitinase Gene Expression Assays

Total RNA was extracted from the mycelium of *T. atroviride* strain CMU-08 following the method described by Gruber et al. [63] during independent confrontation assays against *B. cinerea* and *Fusarium* sp. Samples were collected before contact (BC), during contact (C), and after contact (AC) with these phytopathogens. The extracted RNA was treated with DNase I Amplification Grade (Invitrogen, Carlsbad, CA, USA). Subsequently, 1 μL of oligo (dT)12-18 at 500 μg/mL was added and heated at 70 °C for 10 min. cDNA synthesis was performed using the RevertAid Reverse Transcriptase kit (Thermo Scientific™, Waltham, MA, USA).

Real-time qPCR assays were conducted using the following reaction mixture per reaction: 5 μL iQTM SYBR^®^ Green Supermix (Bio-Rad, Hercules, CA, USA), 30 ng of cDNA template, and 1 μL of each of the oligonucleotides (1.5 μM, Table 2) for a total volume of 10 μL. The amplification protocol included an initial cycle at 95 °C for 5 min, followed by 40 cycles at 95 °C for 15 s, 60 °C for 1 min, and 72 °C for 20 s. The CFX96 Real-Time PCR detection system (Bio-Rad, USA) was used for thermocycling. All reactions were performed in triplicate.

For the relative expression analysis, the average threshold cycle (CT) of the target genes was normalized to the CT of the glyceraldehyde-3-phosphate dehydrogenase (*gpd*) gene and calculated according to the 2^−ΔΔCT^ method [64].

### 2.13. Phylogenetic and Structural Analyses of the Chitinase Encoded in the TRIATDRAFT_217415 Locus of T. atroviride

For the phylogenetic analysis of the protein encoded in the TRIATDRAFT_217415 locus gene, chitinase sequences from *Trichoderma* spp. assigned to groups A, B, and C by previous phylogenetic analyses [65,66,67] were retrieved from Genbank (https://www.ncbi.nlm.nih.gov/). This analysis includes bacterial chitinases acquired by *Trichoderma* spp. through horizontal gene transfer (HGT), orthologous protein sequences from the bacterial taxa, which grouped with group B fungal chitinases, were also included [66]. Selected sequences are listed in Supplementary Table S2. A Fasta file was generated with the selected sequences, and a multiple alignment was performed using the MAFFT server with the default parameters [68]. The resulting alignment was optimized using GUIDENCE2 [69], and the output Fasta file from this optimized alignment was used to determine the best evolutionary model using the IQ-TREE server [70,71].

The phylogenetic tree was constructed using the maximum likelihood (ML) criterion on the IQ-TREE server, employing the WAG + F + I + G4 evolutionary model, and support values for branches were obtained using SH-aLRT support (%)/aBayes support/ultrafast bootstrap (UFBoot) after 1000 iterations [72]. The resulting phylogenetic tree was visualized and edited using iTOL [73].

The structural analysis of the protein encoded in the TRIATDRAFT_217415 locus of *T*. *atroviride* was conducted using the SWISS-MODEL [74], I-TASSER [75], and Phyre2 [76] servers, which generated the 3D model of the putative chitinase.

### 2.14. Statistical Analysis

Statistical analyses were conducted using jmp^®^ (Miami, FL, USA). Data are presented as the mean ± standard deviation or standard error (S.E.), as appropriate for each experiment. Statistical significance for all tests performed was set at *p* < 0.05.

## 3. Results

### 3.1. Growth Kinetics

**Figure 1:**
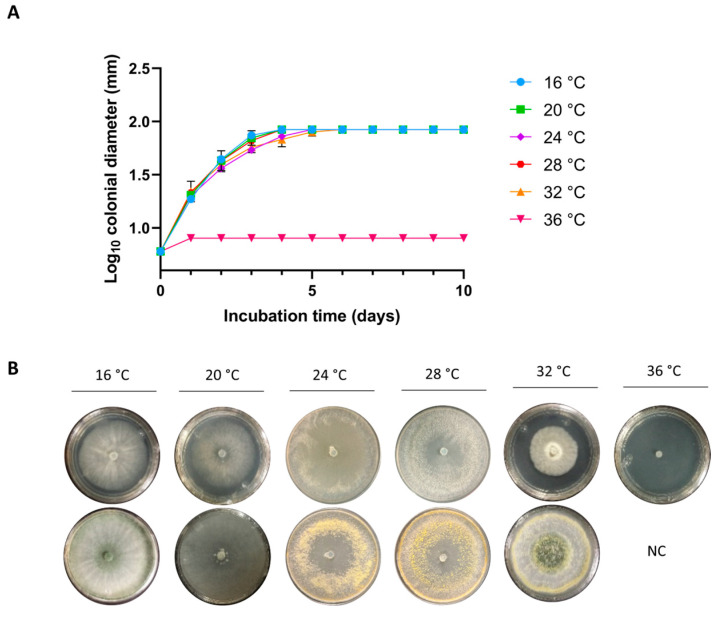
Growth and conidiation of *T. atroviride* strain CMU-08. The strain was inoculated in potato dextrose agar (PDA) medium and incubated at the indicated temperatures. (**A**) For growth kinetics, the colonial diameter was measured every 24 h. The bars represent the standard deviation; in some cases, the bars are small and, therefore, hidden by the growth kinetics symbols. (**B**) In each culture condition, the appearance of the colony before conidiation (plates on the above at each temperature) and the onset of the conidiation process (plates on the below at each temperature) are shown. NC indicates that the strain did not conidiate. The assays were performed in triplicate.

Growth kinetics were conducted to assess the growth and conidiation capacity of the study strain in two different culture media under varying temperature and pH conditions. No significant differences were observed in the growth of strain CMU-08 at temperatures of 16, 20, and 28 °C in PDA. After five days of incubation, the strain entered the stationary phase at the specified temperatures (Figure 1A). A growth delay was noted at 32 °C, with the strain reaching the stationary phase by the fifth day of incubation, while at 36 °C, minimal growth was observed, which halted quickly. Strain CMU-08 initiated conidiation on the third day at 16, 24, and 32 °C, and on the fifth day at 20 °C (Figure 1B). At 16 and 20 °C, conidiation was denser in the center of the colony, whereas at 24 and 32 °C, the characteristic green ring pattern of *Trichoderma* spp. conidiation was observed. Similar patterns of growth and conidiation were obtained when strain CMU-08 was cultivated in MEA medium (Supplementary Figure S1).

**Figure 2:**
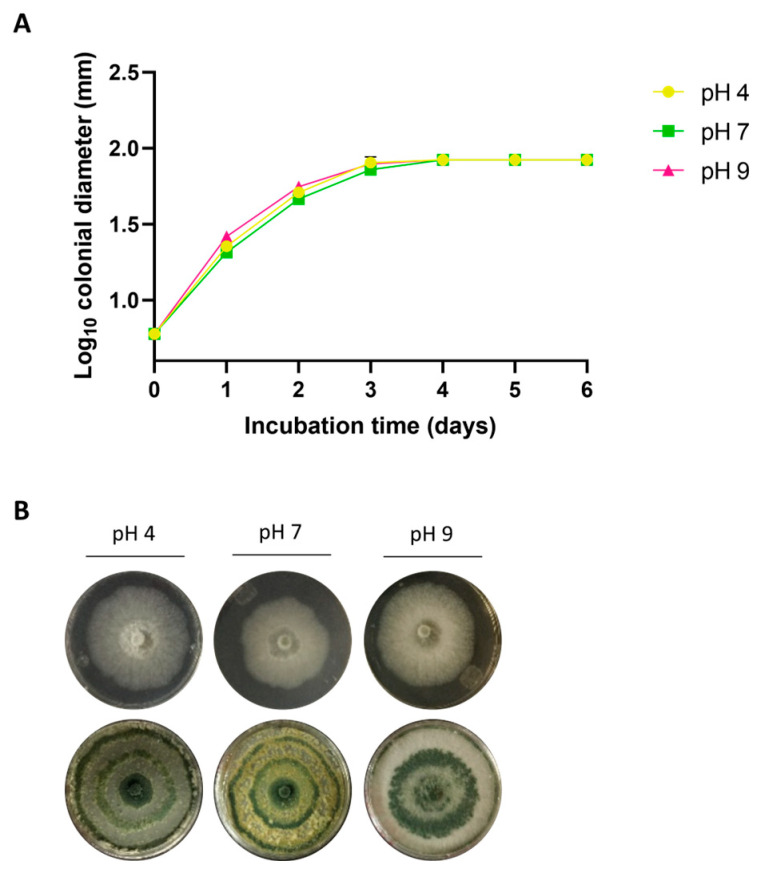
Growth and conidiation of *T. atroviride* strain CMU-08 at different pH levels. Panel (**A**) shows the growth kinetics on potato dextrose agar (PDA) medium. The colonial diameter was measured every 24 h while incubating at 28 °C. Assays were performed in triplicate, and the bars representing the standard deviation are small and, thus, hidden by the growth kinetics symbols. Panel (**B**) illustrates the mycelial development (top row) and the conidiation pattern (bottom row) of the strain under the different growth conditions.

For the assays conducted under different pH conditions, described below, a temperature of 28 °C was selected as optimal for the study strain. CMU-08 covered the surface by the third day of incubation across all three pH conditions evaluated (Figure 2A). At pH 4 and pH 7, conidiation began on the third day of incubation, exhibiting the following similar pattern: starting with a white color in the center of the colony that later turned dark green. At pH 7, conidia also turned yellow, forming rings on the colony. At pH 9, conidia were observed on the second day of incubation in the colony’s center, initially yellow and turning intense green, forming a large ring (Figure 2B). Additionally, it demonstrated similar growth and conidiation in MEA medium at the three pH values tested (Supplementary Figure S2).

### 3.2. Antagonism Assays

#### Antagonism Assays in Dual Culture

**Figure 3:**
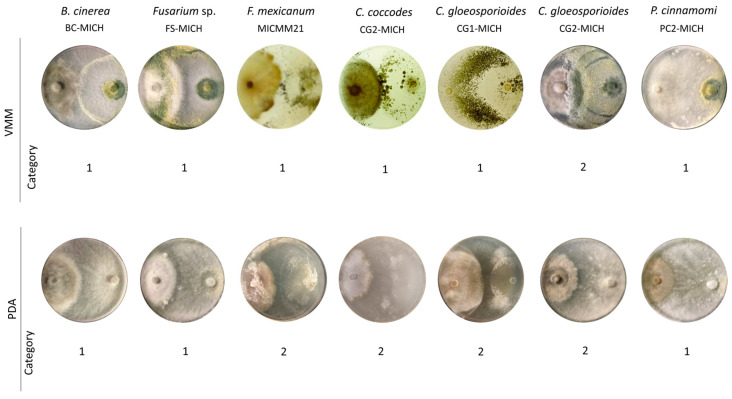
Antagonism in a dual culture of *T. atroviride* strain CMU-08 toward phytopathogenic microorganisms. On the left are the abbreviations of the culture media used, and at the top are the phytopathogens with which the *T. atroviride* strain was tested. According to Worasatit et al. [59], the level of antagonism is indicated at the bottom. In all tests, the CMU-08 strain was inoculated on the right side of the Petri dish and the phytopathogen on the left side. All tests were carried out at a temperature of 28 °C. Key for culture media: VMM, Vogel minimal medium; PDA, potato dextrose agar.

The study strain exhibited the highest level of antagonism (level 1) against three out of the seven phytopathogens tested, while level 2 antagonism was observed against *C. gloeosporioides* strain CG2-MICH across both VMM and PDA media (Table 3, Figure 3). For *F. mexicanum* (MICMM21), *C. coccodes* (CCJT-1), and *C. gloeosporioides* (CCJT-1), the study strain demonstrated level 2 antagonism in PDA medium (Figure 3). The levels of antagonism observed against the phytopathogens were consistent when the assays were conducted in MEA medium (Supplementary Figure S3 and Table S1).

### 3.3. In Vitro Inhibition Assays

#### 3.3.1. Inhibition by Non-Volatile Metabolites

**Figure 4:**
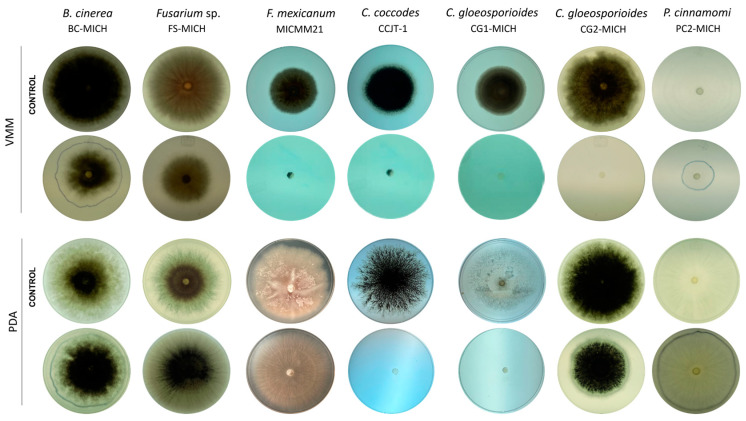
Inhibition assays of phytopathogenic microorganisms by non-volatile metabolites of *T. atroviride* strain CMU-08. In some of the tests, the border of the colony is faintly highlighted with a blue marker, which could not be made visible in the photograph. The rows of control plates for each phytopathogen, without *T. atroviride*, are indicated on the left. All tests were carried out at a temperature of 28 °C. The names of the test phytopathogens appear at the top. Key for the culture media: VMM, Vogel minimal medium; PDA, potato dextrose agar. The assays were performed in triplicate.

In VMM, strain CMU-08 exhibited class 1 inhibition levels against both strains of *C. gloeosporioides* analyzed, as well as against *C. coccodes* and *P. cinnamomi* strains, with inhibition values ranging from 70% to 100%. In this culture medium, class 3 inhibition was observed against *Fusarium* sp., *Fusarium mexicanum*, and *B. cinerea* strains (Table 4, Figure 4). In PDA medium, the study strain demonstrated class 1 inhibition against *C. coccodes* (91.67%) and the *C. gloeosporioides* CG1-MICH strain (93.02%). However, class 2 inhibition (38.09%) was observed against the *C. gloeosporioides* CG2-MICH strain (Table 4, Figure 4). In MEA medium, class 1 inhibitions were observed only against *C. coccodes* (92.68%) and *C. gloeosporioides* CG1-MICH (78.23%) strains (Supplementary Figure S3 and Table S1).

#### 3.3.2. Inhibition by Volatile Metabolites (VOCs)

**Figure 5:**
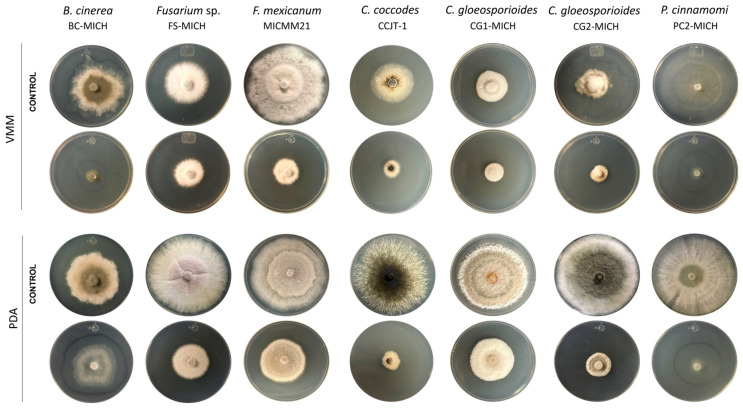
Inhibition of phytopathogenic microorganisms by volatile metabolites (VOCs) emitted by the CMU-08 *T. atroviride* strain. The left side shows the culture media in which the test was carried out, and at the top, the names of the phytopathogenic species tested are indicated. In each culture medium, the row of the control plate is indicated, in which each phytopathogen was incubated without the presence of the CMU-08 strain. All tests were carried out at a temperature of 28 °C. For some of the tests, the outline of the colony is shown faintly with a blue marker to make the level of growth evident. The assays were performed in triplicate. Key for the culture media: VMM, Vogel minimal medium; PDA, potato dextrose agar. For details, see Section 2.

Variations in the inhibition of phytopathogens by volatile metabolites from strain CMU-08 were observed depending on the specific phytopathogen and the culture medium used (Figure 5). In the VMM medium, only the *P. cinnamomi* strain PC2-MICH exhibited class 1 inhibition, with an inhibition percentage of 70.18%. Other phytopathogens tested showed class 2 inhibition in this medium, with inhibition percentages ranging from 50% to 63% (Table 5).

In the PDA medium, volatile metabolites from the CMU-08 strain inhibited the growth of *C. gloeosporioides* strain CG2-MICH by 68.65%, corresponding to class 1 inhibition (Figure 5). Class 2 inhibition was observed for five of the studied phytopathogens, with the percentages ranging from 46.42 to 56.41% (Table 5). The lowest percentage of inhibition (27.24%) was noted against the phytopathogen *F. mexicanum*, classified as class 3 inhibition. In the MEA medium, *B. cinerea* (67.06%) and *P. cinnamomi* (70.23%) strains exhibited class 1 inhibition (Supplementary Figure S3 and Table S1).

In all antagonism tests between the CMU-08 strain and different phytopathogens, whether through dual-culture confrontations, inhibition by non-volatile metabolites, or VOCs, the analysis of variance indicated significant effects of the growth medium used (*p* < 0.05), and the species or strain of the pathogen (*p* < 0.05) on the antagonism outcomes.

### 3.4. Mycoparasitism-Related Structures

**Figure 6:**
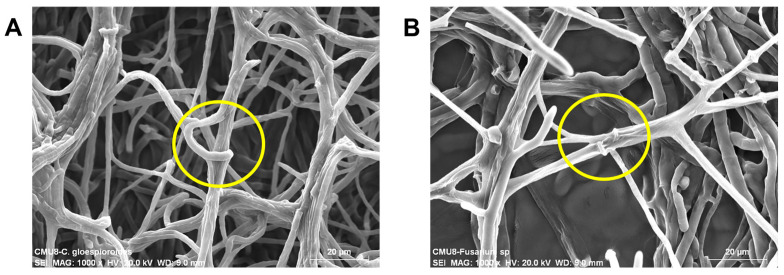
Coiling structures of the mycelium of the *T. atroviride* strain CMU-08 on the mycelium of two test phytopathogens. Interactions with *C. gloeosporioides* (**A**) and *Fusarium* sp. (**B**) are shown. The coils of the CMU-08 strain on each phytopathogen are indicated within the circles.

Photomicrographs obtained during the interaction of *T. atroviride* strain CMU-08 with the test phytopathogen strains in dual cultures revealed the coiling of *T. atroviride* mycelium around the mycelium of each phytopathogen. The hyphae of *T. atroviride* can be distinguished by their thinner appearance and the coiling around the hyphae of phytopathogens, a characteristic of mycoparasitism (Figure 6).

### 3.5. In Vitro Inhibition Assays of B. cinerea by Extracellular Filtrates of T. atroviride Strain CMU-08

Tests were conducted to evaluate the inhibition capacity of secondary metabolites secreted by *T. atroviride* into the culture medium, where extracellular filtrates from the sixth day of incubation of strain CMU-08 were added to Petri dishes containing culture medium with *B. cinerea*. Both basal (BC) and induced (IC) filtrates inhibited the growth of *B. cinerea*. Filtrates at a 5% (*v*/*v*) concentration showed less than 10% inhibition. However, at a concentration of 10% (*v*/*v*), significant inhibition of phytopathogen growth was observed, with 19.36% inhibition for IC and 26.08% for BC (*p* < 0.05) (Figure S4). The same concentration of the concentrated basal filtrate (10% *v*/*v*) effectively inhibited phytopathogen growth, achieving 91.3% inhibition (Figure S4).

**Figure 7:**
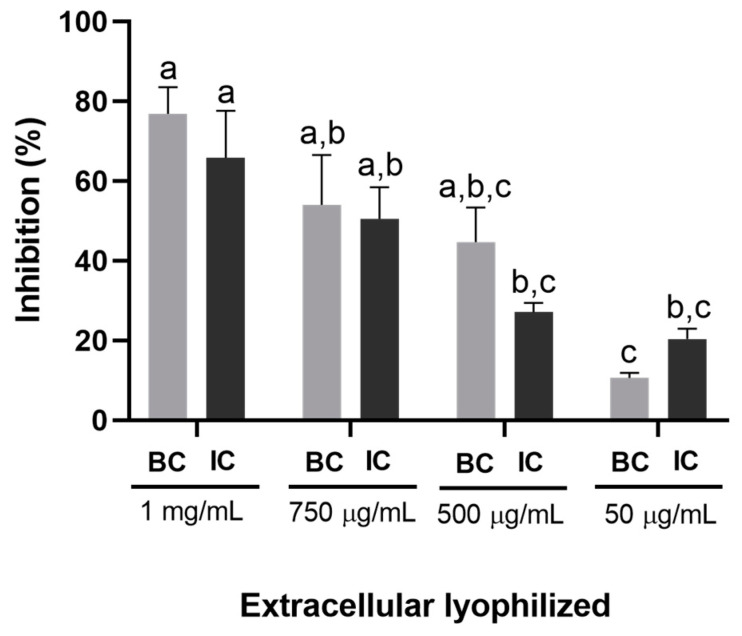
Inhibition of the growth of *B. cinerea* by extracellular filtrates of *T. atroviride* strain CMU-08 in a microplate assay. The figure shows the inhibition percentages of *B. cinerea* growth resulting from the addition of the extracellular lyophilized medium of strain CMU-08. Key for the culture media: BC, basal condition; IC, induced condition. Three independent assays were performed in triplicate. Bars represent the standard error, and different letters indicate significant differences (two-way ANOVA with Tukey’s post hoc test, *p* < 0.05).

Microplate tests with lyophilized extracellular samples of *T. atroviride* strain CMU-08 showed no significant difference in *B. cinerea* inhibition between lyophilized samples from BC and IC conditions (Figure 7). The highest inhibition percentages were 76.8% and 65.8% at 1 mg/mL concentration for basal and induced conditions, respectively. However, a significant difference (*p* < 0.05) was observed among the same lyophilized sample concentrations. Basal lyophilizate similarly inhibited *B. cinerea* at concentrations of 750 (76.8%), 500 µg/mL (54.0%), and 1 mg/mL (44.6%). IC lyophilizate significantly inhibited *B. cinerea* at 1 mg/mL (65.8%) and 750 µg/mL (50.5%) (Figure 7).

A linear regression analysis was conducted using the effects of different tested concentrations to determine the LD_50_ of the extracellular lyophilizates from the strain used to inhibit *B. cinerea*. Figure S5 depicts the R^2^ values and the regression equations used to establish the LD_50_ values for the extracellular lyophilizates under the basal and induced conditions, which resulted in 625.8 and 759.6 µg/mL, respectively.

### 3.6. Reduction in Leaf Damage in Tomato Leaf Assays

**Figure 8:**
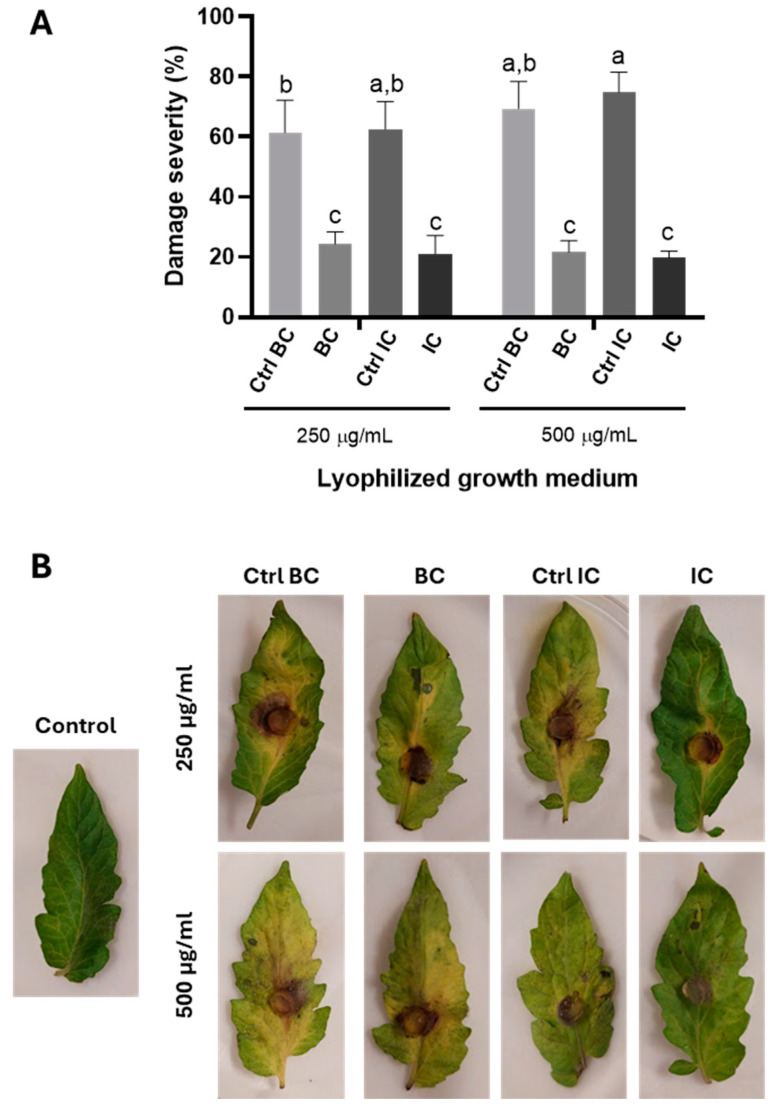
Reduction in the damage severity by *B. cinerea* to tomato leaves treated with the extracellular lyophilized *T. atroviride* growth medium. (**A**) Percentages of the reduction in the severity of damage in tomato leaves inoculated with *B. cinerea* and treated with the extracellular lyophilized strain CMU-08. (**B**) Representative examples of the damage caused by the phytopathogen to tomato leaves treated with the lyophilized *T. atroviride* and their respective controls. The lyophilized cultures of the study strain were evaluated under basal (BC) and induced (IC) culture conditions and their respective uninoculated controls (CtrlBC/CtrllC). Two independent assays were performed in triplicate. Bars represent the standard deviation, and different letters represent significant differences (two-way ANOVA with Tukey’s post hoc test, *p* < 0.05).

**Figure 9:**
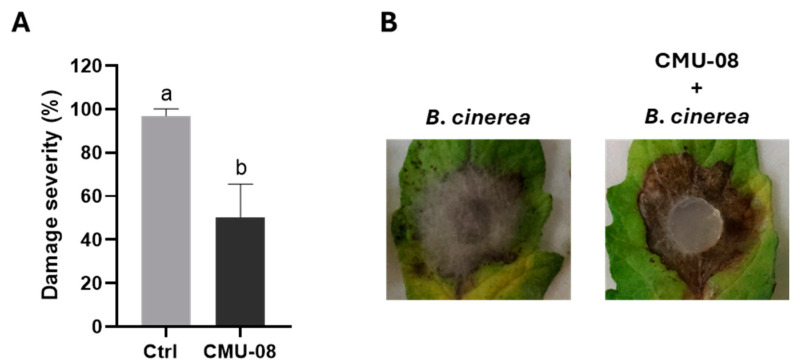
Reduction in the damage severity by *B. cinerea* to tomato leaves and phytopathogen mycelial growth inhibition exposed to volatile metabolites (VOCs) emitted by the CMU-08 strain of *T. atroviride*. (**A**) Percentage of the severity of leaves infected with *B. cinerea* and treated with the VOCs of the CMU-08 strain growing in VMM. (**B**) Representative examples of leaves that show the damage by the phytopathogen in leaves untreated with the VOCs of *T. atroviride* and the effect of such metabolites on the mycelium of the phytopathogen. Bars represent the standard deviation, and different letters represent significant differences (Student’s *t*-test, *p* < 0.05).

A significant reduction in foliar damage caused by *B. cinerea* was observed when applying lyophilized extracellular filtrates of the CMU-08 strain obtained using two different culture conditions. The severity of leaf damage with freeze-dried products was only 31.9 and 34.0% when treated with freeze-dried media under basal (BC) and induced (IC) culture conditions compared to the control (Figure 8). Damage using BC lyophilizates at 250 and 500 µg/mL were 24.41 and 21.60%, respectively, while the severity percentages using IC lyophilizates were 21.12 and 19.68% at 250 and 500 µg/mL (Figure 8). VOCs emitted by the CMU-08 strain also demonstrated a significant reduction in damage caused by *B. cinerea* on tomato leaves, as well as inhibition of vegetative mycelium development of the phytopathogen. Leaves treated with VOCs from the *T. atroviride* strain exhibited a severity percentage of 50.35% (Figure 9A), and although quantitative determination of mycelium development reduction from the inoculum was not performed, it was visibly reduced (Figure 9B).

### 3.7. Quantitative Determination of Chitinase Activity

**Figure 10:**
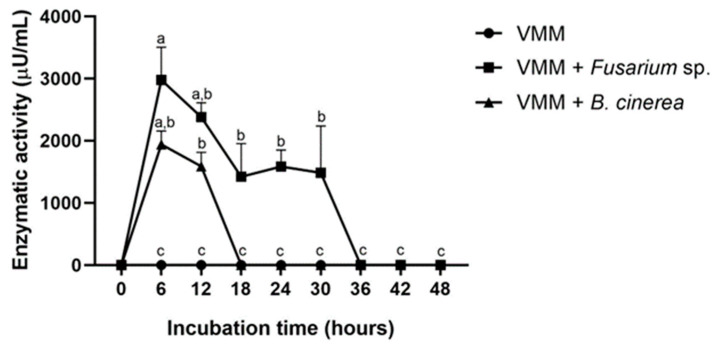
Extracellular chitinase activity of *T. atroviride* strain CMU-08. Chitinase activity is shown under the basal and induction culture conditions with 0.5% (*w*/*v*) *B. cinerea* and *Fusarium* sp. cell walls. The cultures were incubated at 28 °C and 120 rpm in three independent assays in triplicate. Bars represent the standard error, and different letters represent significant differences (two-way ANOVA with Tukey’s post hoc test, *p* < 0.05).

Basal enzymatic activity of chitinase was not detected in *T. atroviride* strain CMU-08 growing on VMM. However, enzymatic activities were observed upon supplementing the medium with *B. cinerea* and *Fusarium* sp. cell walls (Figure 10).

Chitinase activity was detected and reached maximum levels six hours after incubation in the medium supplemented with the cell walls of either *B. cinerea* or *Fusarium* sp. (Figure 10). Maximum chitinase activity induced by *B. cinerea* cell walls was 1941.127 (±216.03) µU/μL, while with *Fusarium* sp. it was 2979.864 (±524.19) µU/μL. In the case of *Fusarium* sp., the activity decreased from 6 to 18 h and remained stable between 18 and 30 h of incubation before declining abruptly at around 36 h. Conversely, with *B. cinerea* cell walls, the enzymatic activity decreased rapidly after 12 h of incubation, becoming undetectable by 18 h.

### 3.8. Expression of Chitinase Genes in CMU-08 Strain

**Figure 11:**
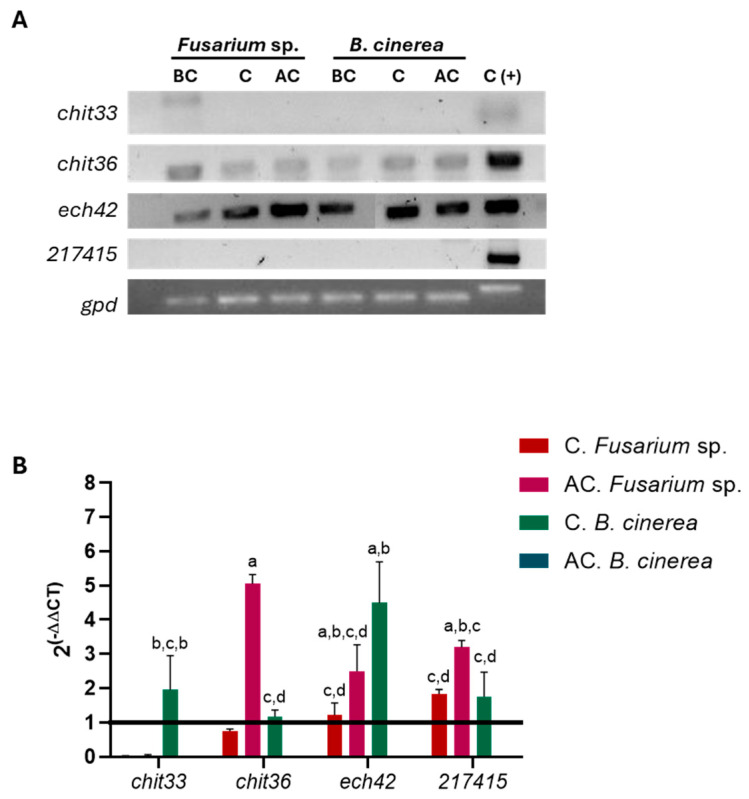
Transcription patterns of chitinase genes from the *T. atroviride* CMU-08 strain. The amplification patterns originating from the transcripts of the *chit33*, *chit36*, *ech42*, and *217415* genes encoding chitinases and the reference gene *gpd* (glyceraldehyde-3-phosphate dehydrogenase) are shown. The amplification products come from the cDNA originated by the total RNA isolated from the mycelium of the CMU-08 strain interacting in a dual culture before contact (BC), during contact (C), and after contact (AC) with the mycelium of the mycelial colonies of each phytopathogen in independent experiments. (**A**) The results of a representative assay from three independent experiments are shown. (**B**) Changes in the transcript levels of the genes analyzed during contact (C) and after contact (DC) between the *T. atroviride* strain CMU-08 and *Fusarium* sp. or *B. cinerea* are shown. Bars represent the standard error of three independent trials. Different letters represent significant differences (two-way ANOVA with Tukey’s post hoc test, *p* < 0.05).

The results of the end-point RT-PCR assays indicated variations in the transcription levels of the genes encoding chitinases in the CMU-08 strain during its interaction with *Fusarium* sp. and *B. cinerea* (Figure 11A).

Quantitative evaluation by RT-qPCR of the gene expression corroborated the findings of the endpoint PCR assay. The *chit33* gene exhibited significant transcriptional activity only during contact with *B. cinerea* mycelium (Figure 11B). The transcription of the *chit36* gene was suppressed during contact with *Fusarium* sp., but its transcript levels markedly increased post-contact with this phytopathogen. In contrast, transcription of this gene was induced solely during contact with *B. cinerea*, followed by transcriptional repression post-contact (Figure 11B). The *ech42* gene showed induced transcription during and after contact with *Fusarium* sp. mycelium, peaking during the latter phase of the interaction process.

Similarly, transcription of this gene was significantly induced during contact with *B. cinerea* but repressed after the physical contact between the mycelia. Transcription of the *217415* gene was induced during and after contact with *Fusarium* sp., with higher expression observed during the initial contact. This gene also exhibited induction of transcription during contact with *B. cinerea* (Figure 11B).

### 3.9. Phylogenetic and Structural Analyses of the Chitinase Encoded in the TRIATDRAFT_217415 Locus

**Figure 12:**
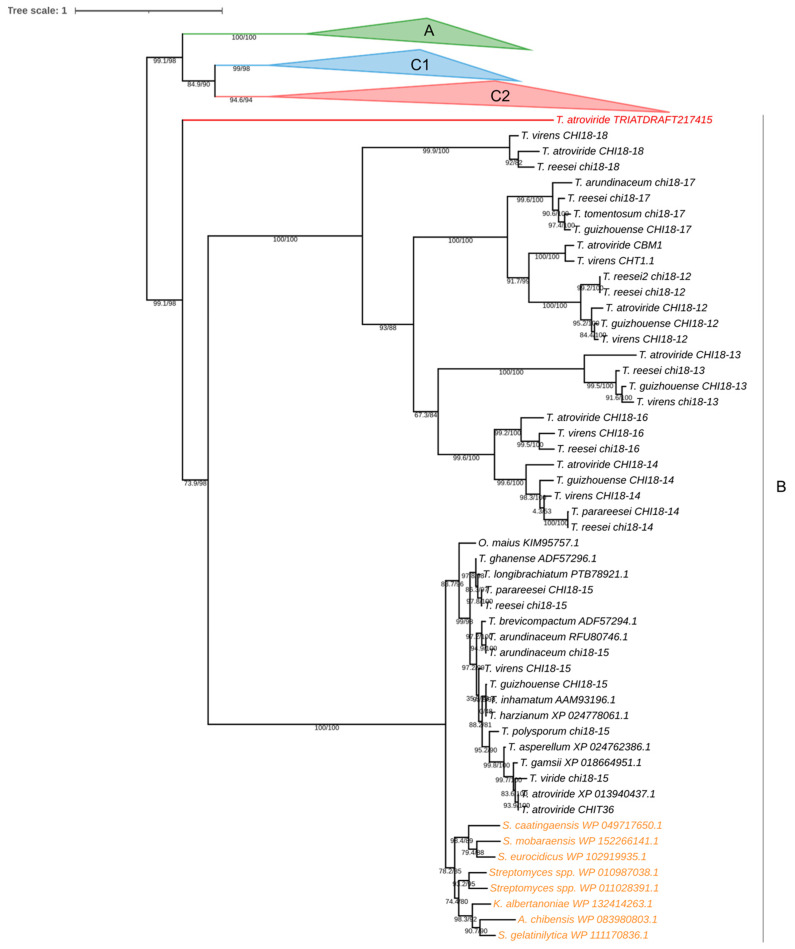
Phylogenetic analysis of the chitinase encoded in the TRIATDRAFT_217415 locus of *Trichoderma atroviride*. The tree was generated using the maximum likelihood criterion with the WAG + F + I + G4 evolutionary model. The terminal branch in which the chitinase of interest appears is shown in red. The terminal branches that group subgroups A, C1, and C2 chitinases are collapsed. The rest of the branches are from subgroup B. Bacterial chitinases related with horizontal transfer to fungi are highlighted in brown. aBayes ultrafast bootstrap (UFBoot) support values obtained after 1000 iterations are indicated in each bifurcation. For details, see Section 2.

Of the chitinases analyzed in the present work, there are no previous experimental or bioinformatics studies on the enzyme encoded in TRIATDRAFT_217415 locus. Because of the transcriptional regulation pattern observed here during the interaction between the CMU-08 strain and the test phytopathogens, we were interested in further characterization of the putative protein encoded in this gene. The protein encoded by the TRIATDRAFT_217415 locus clusters with group B chitinases from *Trichoderma* spp., including orthologs from the bacterial species *Kribbella albertanoniae*, *Actinomadura chibensis*, *Spongiactinospora gelatinilytica*, and *Streptomyces* spp. (Figure 12). However, it splits as an independent branch from the rest of the chitinases within the B clade.

**Figure 13:**
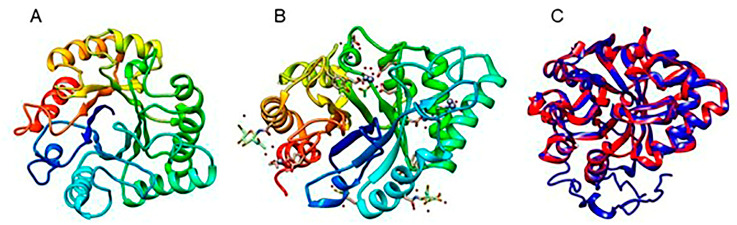
Model of the chitinase encoded in the TRIATDRAFT_217415 locus of *Trichoderma atroviride*. (**A**) The model was obtained with the Phyre2 server. (**B**) 3D configuration of endo-beta-N-acetylglucosaminidase from *Trichoderma reesei* (PDB: 4AC1), which was the best template obtained with the Phyre2 and I-TASSER servers. (**C**) Superimposition of the model generated (structure in blue) with I-TASSER and *T. reesei* chitinase (structure in red, PDB: 4AC1).

Robust 3D models of the hypothetical chitinase encoded by the TRIATDRAFT_217415 locus were generated using bioinformatics tools from Phyre2 and I-TASSER servers, with the crystallized endo-beta-N-acetylglucosaminidase from *Trichoderma reesei* selected as the best template (Figure 13). Additionally, the SWISS-MODEL server utilized a 3D model of an endo-N-acetyl-beta-D-glucosaminidase from *Trichoderma guizhouense* generated with AlphaFold2 as a template (Figure S6). The quality parameters and templates used by these servers are detailed in Figures S7 and S8.

## 4. Discussion

Despite the extensive research conducted on the antagonistic and biocontrol capacities of various species within the genus *Trichoderma*, the imperatives of maintaining or enhancing global agricultural production amidst climate change, the escalating resistance of phytopathogenic fungi to fungicides, the diverse environmental conditions affecting the efficacy of biocontrol strains, and the significant phenotypic and genotypic variability among phytopathogens underscore the ongoing need to isolate and characterize new strains of *Trichoderma* spp.

This study characterized a strain of *T. atroviride* isolated from central Mexico, focusing on its in vitro antagonism against phytopathogen strains isolated from local crops. Environmental factors, such as temperature, pH, and the availability of organic nutrients, profoundly influence conidiation, germination, vegetative growth, and the antagonistic capacity of *T. atroviride* [77,78,79]. Therefore, this work evaluated the effects of these factors on the physiology of the CMU-08 strain.

The choice of culture medium significantly influences the outcomes of in vitro physiological studies on *Trichoderma* spp., particularly those related to antagonism and biocontrol efficacy. Therefore, the physiological and antagonistic characterization of the *T. atroviride* CMU-08 strain was conducted using complete PDA and MEA and the supplemented MMV media to assess their impacts on strain performance.

The PDA medium is well-suited for isolating *Trichoderma* spp. strains and has been extensively utilized to evaluate *T. atroviride*’s antagonism against phytopathogens in dual-culture confrontation assays [80,81,82,83,84]. Recent studies have also highlighted *T. atroviride*’s ability to stimulate the growth and production of lateral roots in *Arabidopsis thaliana* when grown on PDA medium [84]. In early experiments, the MEA medium was initially employed to inhibit phytopathogen growth through volatile and non-volatile metabolites produced by *Trichoderma* spp. [58,85]. However, except for specific cases [86,87,88], MEA is rarely used in growth inhibition or antagonism assays. On the other hand, MMV is suitable for analyzing cellular and molecular processes in *Trichoderma* spp., making it a valuable model for studying mycoparasitism, e.g., [89,90,91].

Analyzing the development and antagonism of the CMU-08 strain across different culture media in this study reflects the varied nutritional environments encountered by phytopathogens targeted for antagonism by *Trichoderma* spp. [92,93,94]. Differences in nutrient composition can be associated with differences in growth and conidiation of strain CMU-08 in the complete culture media used here. These differences were more marked in MEA medium under neutral and alkaline pH conditions. In *T. harzianum*, growth and conidiation are inhibited at alkaline pH in a medium supplemented with ammonium, which is possibly due to the increase in intracellular pH due to internalization through the passive transport of said nitrogen source [95]. Furthermore, the growth and conidiation of *T. harzianum* are also inhibited at pH 7.5 using glutamine or nitrate as a nitrogen source. These interactions between carbon and nitrogen sources with the initial pH of the culture medium may be occurring in the case of the CMU-08 strain, particularly in the MEA medium. Although maltose is a carbon source efficiently used for mycelial development and conidiation of *T. atroviride* [96,97,98], it is possible that other components of the MEA medium, in combination with non-acidic pH, inhibit conidiation. At the molecular level, the environmental factors described above may be influencing the mitogen-activated protein kinase (MAPK) signaling cascades. Tmk1 MAP kinase is involved in the control of vegetative growth and conidiation in *T. atroviride* [99]. It is necessary to carry out studies at this level in the CMU-08 strain and other *T. atroviride* strains to evaluate how conserved the expression patterns of these signaling factors are under different environmental conditions.

The *T. atroviride* strain CMU-08 exhibited optimal growth and varying efficiency in conidiation across PDA and MEA media at all evaluated incubation temperatures. Similar physiological characteristics were observed in other *T. atroviride* strains, such as strain T-15603.1, which showed no significant differences in growth between MEA and nutrient-limited LNA media at temperatures ranging from 25 to 30 °C [45,80]. For the LU132 strain, enhanced conidial production was observed in a culture medium with a 160:1 carbon-to-nitrogen ratio, supplemented with sucrose as the carbon source, and incubated at 25 °C for 20 days [77,78]. Although both PDA and MEA media contain dextrin derived from starch hydrolysis, MEA also features a high concentration of maltose, whereas PDA is supplemented with dextrose, representing a fundamental nutritional difference. The composition of PDA was initially analyzed for fungal growth, revealing that nitrate and ammonium serve as nitrogen sources, in addition to organic acids and various amino acids [100]. Although the precise mineral micronutrient content of the PDA and MEA media was not provided by the suppliers, variations in micronutrients are likely due to differences in the origin of the extracts used. Therefore, PDA and MEA offer complementary data in the characterization of *Trichoderma* spp. strains. Documented physiological differences when mycelial fungi grow in both media include growth inhibition of the mycelium by plant extracts [101], qualitative and quantitative differences in the production of soluble and volatile secondary metabolites [102,103], and toxin production [104], among others. Despite this, studies comparing the physiology of new *Trichoderma* spp. strains in both media are lacking. Thus, when possible, the characterization of *Trichoderma* strains should be considered using both PDA and MEA media.

The experiments in a solid medium that evaluate the effect of pH on *Trichoderma* spp. can be performed in a buffered or non-buffered medium, generating relevant physiological information in both cases [95]. It has been proposed that, unlike media with buffered pH, in the non-buffered medium light stimulates the intracellular acidification of the mycelium of *Trichoderma* spp., favoring conidiation. In the PDA medium, the CMU-08 strain grew and conidiated under the three conditions of the initial pH evaluated (acidic, neutral, and alkaline), while in the MEA medium, it grew under all three conditions but produced conidia only in the acidic pH. Our results suggest that regardless of the initial pH, in PDA the strain can acidify the medium, achieving conidiation, although modifying the conidiation pattern at alkaline pH. In contrast, it is possible that in MEA the studied strain cannot acidify the medium at a neutral or alkaline pH where it grows but is unable to conidiate. These results do not agree with previous studies carried out in non-buffered media, in which it has been observed that the initial pH of the culture medium that favors mycelial growth also favors the conidiation process, with an optimal pH between 4.0 and 6.8. Furthermore, *T. atroviride* strain LU132 significantly decreased its conidiation capacity in PDA medium as the pH increased, being unable to conidiate at a pH higher than 7.0 [77].

In addition to its contribution to the basic physiological knowledge of *T. atroviride*, the analysis of the growth and conidiation patterns in culture media with non-buffered pH has direct biotechnological implications. Thus, unlike *T. harzianum*, *T. atroviride* cannot develop efficiently in savoy cabbage and rapeseed straw composts with an initial alkaline pH of 8.8, which could be used as a propagation medium for a biocontrol product [105]. The low survival of three strains of *T. atroviride* in these composts was observed even at high inoculation densities (10^6^ g^−1^), and significant intraspecific differences were observed in the ability to reduce the initial pH of the compost. In *T. harzianum*, the *pac1* gene is a transcriptional regulator that responds to external pH, which is associated with growth and conidiation, while it regulates the expression of genes associated with mycoparasitism, including *chit42* chitinase, *papA* protease, *gtt1* glucose permease, and *qid74* cell wall protein [95]. Further studies are necessary to analyze whether the *T. atroviride* homolog performs similar functions depending on the incubation pH. All the above shows that obtaining basic physiological information and biotechnological use is complemented by the study in non-buffered and buffered pH media; so in the future, it is necessary to analyze the physiology of the CMU-08 strain in the last medium.

The growth and conidiation experiments with the CMU-08 strain were carried out in the dark, with light pulses every 24 h, so it is necessary to evaluate its growth and conidiation under different lighting conditions, as has been tested for the reference strains of *T. atroviride* [106]. In the physiological characterization of strain LU298, the choice of culture media highlighted the relevance of the photoperiod for the mechanical damage or brief light pulses when grown on PDA medium [107]. Additionally, conidiation in response to light pulses and mechanical damage increased under acidic pH conditions between 2.8 and 3.6 [107]. Furthermore, supplementing a minimal medium with organic and inorganic nitrogen sources promoted conidiation under acidic pH, especially in response to mechanical damage [106]. The ability of the CMU-08 strain to grow in two different media within the evaluated pH and temperature ranges is noteworthy. To our knowledge, the physiological versatility of the CMU-08 strain has not been previously described for other *T. atroviride* strains but further studies are needed to fully understand the biochemical and genetic basis of this distinctive trait by the continued study of strains like LU298, LU132, and CMU-08 across different geographic isolates of *T. atroviride* to further document the species’ physiological adaptability and regional variations. In this study, in vitro tests were conducted to assess the CMU-08 strain’s ability to antagonize various phytopathogen strains affecting crops in the same geographical region. Confrontation assays in a dual culture and evaluation of the volatile inhibition were employed to generate hypotheses regarding the mechanisms underlying its antagonistic effects. The results demonstrate variability in the CMU-08 strain’s antagonistic capacity against different phytopathogens tested. Dual-culture confrontation assays provided evidence of the CMU-08 strain’s mycoparasitic potential, which involves direct attack mechanisms, such as the production and secretion of lytic enzymes like chitinases, glucanases, and proteases [34,108]. Additionally, *Trichoderma* spp. employ antibiosis as another mechanism to antagonize phytopathogens, secreting secondary metabolites that are either soluble or non-volatile (NVMs) and volatile (VOCs), effectively inhibiting the growth of phytopathogens [109,110,111].

The CMU-08 strain exhibited Type 1 antagonism at the highest level in MMV against six of the studied phytopathogens, whereas in PDA and AEM, this level of antagonism was observed for only three phytopathogens. Tests in these two culture media revealed Type 2 antagonism against the remaining phytopathogens. These results suggest that the CMU-08 strain is an effective antagonist against phytopathogens under restrictive nutritional conditions (MMV) and non-restrictive conditions (PDA and AEM), albeit less efficiently in the latter. The cost-effective and straightforward characterization approach provides initial insights into the physiological versatility of the strain, laying a foundation for scaling up the cultivation volumes with potential applications in mass production, particularly with complete media. Furthermore, minimal and defined media like MMV enable the study of fundamental growth kinetics and sporulation patterns, facilitating precise biochemical and molecular evaluations. These evaluations are crucial for understanding the effects of different culture variables on development, differentiation, and mycoparasitism processes.

The effects of non-volatile metabolites (NVMs) and volatile organic compounds (VOCs) produced by the CMU-08 strain on the tested phytopathogens were also analyzed. NVMs showed high inhibition percentages against *C. coccodes* and one strain of *C. gloeosporioides* in the cultivation media used. However, *B. cinerea* and the two *Fusarium* spp. strains exhibited the lowest inhibition percentages across all three culture media. In general, inhibition by NVMs and VOCs was less efficient than that observed in the dual confrontation assays, primarily resulting in Classes 2 and 3 inhibitions.

Microphotographs of the contact zone between the CMU-08 strain and the two phytopathogens corroborate its mycoparasitic lifestyle, clearly revealing its coiling around the phytopathogens hyphae. *Trichoderma* secretes enzymes to degrade the phytopathogen’s cell wall during these interactions, synergizing with antifungal secondary metabolites [19,35,112,113,114]. These findings indicate that the CMU-08 strain employs mycoparasitism as its primary antagonist mechanism, potentially complemented by nutrient competition and antibiosis.

Similar mycoparasitic antagonism levels (Type 1 and Type 2) have been reported for other *T. atroviride* strains in dual-culture assays on PDA medium against phytopathogen strains of the same species, including *B. cinerea*, *Fusarium* spp., *C. gloeosporioides*, and *Phytophthora* spp. [113,115,116,117]. However, variations exist among *T. atroviride* strains in their antagonistic effectiveness against phytopathogenic strains of the same species [50,83,118], even among genetically identical *T. atroviride* strains isolated from the same geographic region [48]. Our results align with these previous observations, underscoring that the mycoparasitic efficiency of *T. atroviride* varies depending on the specific phytopathogen species/strain and the composition of the culture medium used in the assay.

During direct confrontation assays, *Trichoderma* spp. secretes hydrolytic enzymes both constitutively and in response to molecules released from the host through enzymatic degradation of its cell wall [119]. On the other hand, the synthesis and release of NVMs and VOCs by *Trichoderma* depend on specific incubation conditions [120]. It is plausible that the three cultures media evaluated in this study may not be optimal for inducing the synthesis of secondary metabolites by the CMU-08 strain. Therefore, future research should optimize incubation conditions to enhance the production of NVMs and VOCs by CMU-08.

The phytopathogen *B. cinerea* is globally significant due to its broad host range and economic impact on numerous crops [121,122]. The strain of this species used in our study exhibited lower susceptibility to inhibition by the CMU-08 strain in various in vitro assays, which is particularly concerning given its agricultural relevance in our geographic region. Additionally, this strain has shown resistance to conventional fungicides (unpublished data). We assessed the impact of the CMU-08 strain’s extracellular filtrates *B. cinerea* growth to address this challenge.

It is important to emphasize the differences observed in the inhibition tests with the extracellular filtrates of strain CMU-08. In non-concentrated extracellular filtrates, some metabolites with antifungal activity may be diluted, particularly when the medium is not supplemented with the walls of the phytopathogen. Conversely, concentrating the filtrates using a rotary evaporator at 70 °C may inactivate some of the antifungal metabolites, while lyophilizing the medium should preserve soluble thermolabile metabolites. Exposing *B. cinerea* mycelium to 10% (*v*/*v*) non-concentrated extracellular filtrates of the CMU-08 strain resulted in up to 50% inhibition in the PDA medium. Similarly, inhibition levels remained consistent at 750 µg/mL concentration across both culture conditions when concentrating and lyophilizing the culture medium supplemented with pathogen cell walls. This similarity suggests either uniform metabolite synthesis or the production of metabolites with similar antifungal efficacy by the CMU-08 strain under different growth conditions.

Previous studies have reported similar inhibitory effects of *T. atroviride* extracellular filtrates on *B. cinerea* growth, although without specifying the concentration of filtrate used [123]. In this previous work, fermentation broth of *T. atroviride* and the hormone brassinolide effectively controlled *B. cinerea* infections in tomato plants, demonstrating superior disease management compared to individual components [123]. Future investigations could explore similar strategies with the CMU-08 strain, potentially leveraging its extracellular metabolites from cost-effective liquid culture to develop novel biocontrol formulations. This approach could enhance efficacy beyond the use of conidia or chlamydospores alone. Moreover, extracellular filtrates and their organic extracts from various *T. atroviride* strains have demonstrated inhibitory effects on other phytopathogens related to those studied here, such as *Fusarium* spp., *Colletotrichum* spp., and *Phytophthora* spp. [50,124].

The extracellular filtrates from both culture conditions of the CMU-08 strain significantly inhibited lesions caused by *B. cinerea* in the tomato leaf assay. In a similar assay using conidia instead of extracellular filtrates, only the LU132 strain out of five tested *T. atroviride* strains showed inconsistent significant inhibition of foliar damage caused by *B. cinerea* in strawberry leaves [120]. However, when extracellular filtrates from the LU132 strain’s fermentation in the Czapek–Dox minimal medium were added, they effectively inhibited the hyphal length of the phytopathogen on strawberry leaves.

In our study, extracellular filtrates from the CMU-08 strain under basal and supplemented with *B. cinerea* walls conditions showed no significant difference in reducing leaf damage. Similar findings were observed with extracellular filtrates from the LU132 strain in axenic culture and co-culture with the phytopathogen strain [120]. These results suggest that soluble metabolite antibiosis is not an efficient antagonism mechanism in the LU132 strain, contrasting with the effective mechanism in the CMU-08 strain. Furthermore, they indicate that neither phytopathogen cell walls nor co-cultivation with them in liquid culture quantitatively or qualitatively alter the extracellular metabolites produced by *T. atroviride*, highlighting the intra-specific physiological variation among geographical isolates of this biocontrol species. Future studies should explore these interactions in greater detail.

Organic extracts from *T. atroviride* mycelium and extracellular filtrates of *T. virens* have demonstrated complete inhibition of *B. cinerea* growth and foliar lesions caused by *Phytophthora capsici* on tomato leaves, respectively [110,111]. However, while organic extraction can isolate compound groups for further study, it introduces complexity and costs to bioformulation production for field use.

Like soluble metabolites, the volatiles (VOCs) produced by the CMU-08 strain significantly inhibit foliar damage caused by *B. cinerea* in tomato plants. Interestingly, the efficacy of VOCs in inhibiting the phytopathogen on leaves exceeds their effectiveness in culture medium, a phenomenon known as “mycofumigation” or specifically “Trichofumigation” for *Trichoderma* species. While studies on *Trichoderma* species have reported mycofumigation effects for various strains of different species within the genus, specific investigations on *T. atroviride* are limited [125].

A recent comparative study between the reference strains P1 and IMI 206040 of *T. atroviride* revealed significant differences in VOC emission patterns, with strain P1 demonstrating superior capacity [43]. In the latter case, the emission of identified VOCs in these strains was associated with responses to abiotic and biotic stresses, particularly the presence of mycotoxins from *F. oxysporum*. Analysis of the VOCs emitted by two *T. atroviride* strains native to New Orleans (USA) identified 39 metabolites, including alcohols, aldehydes, ketones, and esters [126]. The VOC mixture emitted by these strains inhibited the mycelial growth of *Phytophthora infestans* by more than 80%, inducing morphological and cytological damage to the hyphae. Notably, isoamyl and isobutyl alcohols, two major components in the VOC mixture, exhibited complete inhibition of the phytopathogen’s growth.

In another study, a *T. atroviride* strain isolated in Argentina emitted a mixture of seven VOCs that inhibited the growth of *B. cinerea*, with 6-pentyl-α-pyrone showing significant antifungal activity as a single compound [127]. Moreover, post-harvest exposure of blueberries to the VOCs from this strain significantly suppressed phytopathogen development in the fruit. Meanwhile, a closely related species tentatively designated as *Trichoderma* sp. “*atroviride* B” emitted 32 metabolites in its VOC mixture, exhibiting qualitative and quantitative variations in VOC production among the strains studied [128]. Several characterized metabolites in this study inhibited more than 50% of the growth of the phytopathogens, such as *Rhizoctonia solani*, *Alternaria radicina*, *Fusarium oxysporum* f. sp. *lycopersici*, and *Sclerotinia sclerotiorum*. Key compounds included 6-pentyl-pyrone, nerolidol, 2-undecanone, geranylacetone, (+)-limonene, and (−)-limonene.

The inhibition of *B. cinerea* by Trichofumigation in the tomato leaf test can be attributed to some of the metabolites previously described in studies with other *T. atroviride* strains. These studies highlight considerable quantitative and qualitative variations in VOC emissions among the analyzed strains. Interestingly, wild strains have been found to produce higher levels of VOCs compared to reference strains, underscoring the importance of investigating VOC profiles across diverse geographic isolates of *T. atroviride*. Trichofumigation using VOCs from other *Trichoderma* species, such as *T. asperellum*, *T. harzianum*, and *T. koningiopsis*, has also shown protective effects on tomato seedlings and fruits against *B. cinerea* infection [129,130]. Given the CMU-08 strain’s efficiency in inhibiting a local strain of *B. cinerea*, which poses significant threats to regional crops, future research must characterize the VOCs emitted by CMU-08. This will facilitate the evaluation of its potential for application in Trichofumigation processes aimed at protecting various fruits.

Another desirable characteristic in *Trichoderma* spp. strains with potential for biocontrol is their ability to produce extracellular hydrolytic enzymes, particularly those involved in mycoparasitism, pest antagonism, and induction of plant defenses [19,91,131]. Among these enzymes, extracellular chitinases play a crucial role in degrading chitin, a major component of fungal cell walls, and are essential for mycoparasitic activities and nematode antagonism [34,112]. In liquid medium, strain CMU-08 did not exhibit basal extracellular chitinase activity but showed induced activity when the medium was supplemented with cell walls from *B. cinerea* and *Fusarium* sp. The levels of extracellular chitinase activity vary significantly among *Trichoderma* strains and species, both under basal conditions and when induced by phytopathogen cell walls [99,132,133]. Catabolite repression of chitinase activity has been documented in *T. harzianum* but not in the P1 strain of *T. atroviride* [134,135,136]. These differences suggest that transcriptional regulation of chitinase genes in *Trichoderma* species may vary interspecifically and intraspecifically. To further elucidate these differences, we evaluated the transcription levels of genes encoding chitinase activity in the CMU-08 strain.

Transcriptional analysis of the genes *ech42*, *chit33*, *chit36*, and *217415* loci in strain CMU-08 revealed significant variations depending on the phytopathogen species and the stage of interaction during dual-culture confrontation. Such variability in chitinase gene expression is well documented across *Trichoderma* spp. For instance, studies on subgroup C chitinase gene in *T. atroviride* showed distinct expression patterns when by cell walls from *B. cinerea* versus *R. solani*, with eight genes upregulated after 40 h of exposure to the former, compared to only one gene (*tac6*) induced by the latter. Notably, these chitinase genes were overexpressed during and after contact with *B. cinerea* but not *R. solani* [63].

The *ech42* gene, now designated as *chi18-5*, encodes a chitinase belonging to group A, which is widely conserved and extensively studied in fungi, particularly within the Ascomycota. The enzyme produced by *ech42* is notably abundant in the supernatants of induced cultures from the P1 strain *T. atroviride* [67,136,137]. Experimental and bioinformatics evidence indicates its involvement not only in mycoparasitism but also in saprophytism and development processes [67,138]. In the P1 reference strain of *T. atroviride*, *ech42* demonstrates transcriptional induction when exposed to cell walls of *B. cinerea* and *R*. *solani*, under carbon-starve conditions, and in response to various stressors, as well as during dual-culture confrontations with phytopathogens, without evidence of catabolic repression [135,136,139,140,141].

Contrastingly, in CMU-08, transcriptional induction of *ech42* occurs during contact with both test phytopathogens, but with the following distinct dynamics: *Fusarium* sp. induces maximal transcription levels post-contact, whereas *B. cinerea* shows lower levels at this last stage of interaction. Moreover, the magnitude of transcriptional induction varies significantly between confrontations with *S*. *sclerotiorum* mycelium, sclerotia, and apothecia, revealing repression of *ech42* transcription, highlighting interspecific regulatory mechanisms [142].

It is noteworthy that previous studies primarily evaluated *ech42* induction in the P1 strain using Northern hybridization, the end-point RT-PCR, or detection via the *ech42*::GFP construct under fluorescence microscopy. To our knowledge, this study is the first to employ RT-qPCR to precisely assess *ech42* gene transcriptional differences in a *T. atroviride* strain under varying cultivation conditions.

To date, transcriptional regulation studies on the *chit33* and *chit36* genes of *T. atroviride* have not been conducted, although these genes have been investigated in other *Trichoderma* species. The *chit33* gene, now designated as *chi18-12*, encodes a group B chitinase in *T. harzianum* subject to catabolite repression. It is induced under conditions of nitrogen starvation and in response to osmotic and thermal stress. Interestingly, while not significantly induced by chitin or cell walls of *R. solani*, *chit33* shows upregulation during confrontation tests with this phytopathogen and parasitism toward *Caenorhabditis elegans* eggs [143,144]. Transcriptional induction of *chit33* has also been observed during interactions of *T. harzianum* with *S*. *sclerotiorum* mycelium, sclerotia, and apothecia [142].

Previous phylogenetic analysis indicates that the *chit36* gene (*chi18-15*) in *T. atroviride* was likely acquired via horizontal gene transfer from a bacterial ancestor, potentially from the genus *Streptomyces* to a fungal ancestor within the order Hypocreales. Once acquired, *chit36* has undergone selective pressures in *Trichoderma* spp. [145,146]. The *T. asperellum chit36* gene is subject to catabolite repression and is transcriptionally induced under nitrogen starvation, thermal and osmotic stresses, and during interaction with *R. solani* [147]. The CHIT36 enzyme in *T. harzianum* has been shown to inhibit *B. cinerea* conidia germination and the mycelial growth of *F. oxysporum* and *Sclerotium rolfsii* [148].

The phylogenetic and structural analyses conducted on the chitinase encoded by gene *217415* indicate that it belongs to subgroup B. However, such protein split from the rest of the *Trichoderma* subgroup B chitinases included in the phylogenetic analysis, suggesting functional differences with other *Trichoderma* spp. chitinases. This gene has not been previously analyzed in any *Trichoderma* species. Transcription of this gene is induced during interactions with *Fusarium* sp. and *B. cinerea*, with its highest expression levels observed after contact with the former phytopathogen. Such data suggest the involvement of the chitinase encoded by gene *217415* in mycoparasitism. Further studies are necessary to know if such genes have this function in other *Trichoderma* species.

Both the enzymatic activity and transcriptional regulation assays suggest significant intraspecific differences in regulatory mechanisms between the P1 and CMU-08 strains of *T. atroviride*. Significant differences have been found between *T. atroviride* and *T. virens* in the transcriptional regulation of genes associated with mycoparasitism [149]. However, in fungi, these differences can occur among populations of the same species, subject to different selective pressure factors [150,151]. It is necessary to conduct further studies of transcriptional regulation of genes encoding for extracellular hydrolytic enzymes related to mycoparasitism in more *T. atroviride* strains to understand how different the regulation mechanisms could be and what selective pressure factors govern such differences. In a complementary way, the differences in the transcript levels of the *ech42* gene between strain P1 and CMU-08 evaluated in the present work may be due to the different chitin contents of phytopathogens studied and its differential release of probable cell wall inducers, since the tests of the former were carried out in confrontation with *R. solani*, not used in this study. The chitin content of the vegetative mycelium of *B. cinerea* varies between 1 and 5% of the cell wall weight as the incubation time progresses from day 3 to day 9 of incubation, respectively [152]. In contrast, the chitin content in the mycelium of three different *F. oxysporum forma specialis* varied between 8% and 11% [153]. Growth conditions and the method of determination influence these estimates.

## 5. Conclusions

The CMU-08 strain of *T. atroviride* employs diverse antagonistic strategies against various phytopathogen species, prominently utilizing antibiosis through soluble and volatile metabolites alongside mycoparasitism facilitated by multiple extracellular chitinases. The physiological analysis revealed that CMU-08 is an effective antagonist under different nutritional and environmental conditions. This study represents the first documentation of differential expression patterns of the *ech42*, *chit33*, *chit36*, and *217415* genes in a *T. atroviride* strain using RT-qPCR assays during confrontations with *Fusarium* sp. and *B. cinerea*. Similar analysis with different phytopathogens should be extended to other *T. atroviride* strains to ascertain the conservation of these transcriptional regulation patterns in mycoparasitism. These investigations will facilitate the assessment of intraspecific variations during mycoparasitism interactions and contribute to hypothesizing the significance of transcriptional differences in genes involved in fungus–fungus interactions. Moreover, broader evaluations encompassing the transcriptional profiles of chitinase genes from each subgroup will help delineate individual enzymes’ physiological and ecological roles and their impacts on *Trichoderma* spp. lifestyles. Greenhouse trials with the CMU-08 strain are necessary to confirm its other biocontrol attributes, such as promoting plant growth. Omic studies, including genomic, metabolomic, and proteomic analyses, are also in progress. These studies will enable a more detailed molecular evaluation of the physiological versatility and antagonistic properties of the CMU-08 strain. This comprehensive approach will advance the development of formulations for field application and facilitate the eventual commercialization of the CMU-08 strain.

## Figures and Tables

**Table 1:** Phytopathogenic microorganisms used in antagonism assays.

Strain ID	Species	Crop/Plant of Isolation	Locality of Origin ^1^
BC-MICH	*Botrytis cinerea*	Strawberry (*Fragaria × ananassa*)	Uruapan
FS-MICH	*Fusarium* sp.	Blackberry (*Rubus ulmifolius*)	Los Reyes
MICMM21	*Fusarium mexicanum*	Mango (*Mangifera indica*)	Buenavista Tomatlán
CCJT-1	*Colletotrichum coccodes*	Tomato (*Solanum lycopersicum*)	Morelia
CG1-MICH	*Colletotrichum gloeosporioides*	Formio (*Phormium tenax*)	Morelia
CG2-MICH	*Colletotrichum gloeosporioides*	Avocado (*Persea americana*)	Uruapan
PC2-MICH	*Phytophthora cinnamomi*	Avocado (*Persea americana*)	Uruapan

^1^ All localities are within Michoacán State, central Mexico (see Cázares-García et al. [55]).

**Table 2:** Oligonucleotides used for the expression analysis of chitinase and glucanase genes.

Locus(gen)/Oligonucleotide	Sequence (5’→3’) ^1^
*chit33*	
49766RT-Fwd	GCTTCGCCATCGCAGCTGGC
49766RT-Rev	CAGGCCCGACGGGAAGCG
*chit36*	
83999RT-Fwd	CCAAAACGGCCGACTGTGGTGG
83999RT-Rev	GCCCCCGCCAGCTCCATTTTG
*ech42*	
131598RT-Fwd	CCATTGCTGCCCCCGCTGG
131598RT-Rev	GGTCTGGCCAATGCCACCGG
*TRIATDRAFT_217415*	
217415RT-Fwd	CAGCGGCTTCGGCACCATGG
217415RT-Rev	CAGGCCAGGGCGCAGAATCTC

^1^ Oligonucleotides used were validated in the Alfredo Herrera-Estrella lab.

**Table 3:** Antagonism of *Trichoderma atroviride* strain CMU-08 against phytopathogenic microorganisms in dual cultures ^1^.

Phytopathogen (Strain)	Antagonism Level in Each Culture Medium	
	VMM	PDA
*B. cinerea* (BC-MICH)	1 ^a^	1 ^a^
*Fusarium* sp. (FS-MICH)	1 ^a^	1 ^a^
*F. mexicanum* (MICMM21)	1 ^a^	2 ^b^
*C. coccodes* (CCJT-1)	1 ^a^	2 ^b^
*C. gloeosporioides* (CG1-MICH)	1 ^a^	2 ^b^
*C. gloeosporioides* (CG2-MICH)	2 ^b^	2 ^c^
*P. cinnamomi* (PC2-MICH)	1 ^a^	1 ^a^

^1^ The keys and isolation sites of each phytopathogen are shown in Table 1. Antagonism levels were established according to Worasatit et al. [59], with level 1 being the most efficient antagonism of *T. atroviride* strain CMU-08. All tests were carried out at a temperature of 28 °C. Culture media: MMV, Vogel minimal medium; PDA, potato dextrose agar. Different letters represent significant differences (two-way ANOVA with Tukey’s post hoc test, *p* < 0.05). For details, see Section 2.

**Table 4:** Inhibition of the growth of phytopathogenic microorganisms by non-volatile metabolites of the CMU-08 strain of *T. atroviride* ^1^.

Phytopathogen (Strain)	Inhibition Class in Each Culture Medium	
	VMM	PDA
*B. cinerea* (BC-MICH)	3 (31.54) ^b,c^	3 (16.26) ^a,b^
*Fusarium* sp. (FS-MICH)	3 (30.55) ^b,c^	3 (0) ^a^
*F. mexicanum* (MICMM21)	3 (25) ^b,c^	3 (0.41) ^a^
*C. coccodes* (CCJT-1)	1 (100) ^e^	1 (91.67) ^e^
*C. gloeosporioides* (CG1-MICH)	1 (100) ^e^	1 (93.02) ^e^
*C. gloeosporioides* (CG2-MICH)	1 (92.85) ^e^	2 (38.09) ^c^
*P. cinnamomi* (PC2-MICH)	1 (70.23) ^d^	3 (8.73) ^a^

^1^ The keys and isolation sites for each phytopathogen are shown in Table 1. Inhibition classes were established according to Sarven et al. [61], in which class 1 toward the phytopathogen was the most efficient for the CMU-08 strain of *T. atroviride* and class 3 the least efficient. All tests were carried out at a temperature of 28 °C. The inhibition percentages in each case are shown in parentheses. Key for the culture media: VMM, Vogel minimal medium; PDA, potato dextrose agar. Different letters represent significant differences (two-way ANOVA with Tukey’s post hoc test, *p* < 0.05). For details, see Section 2.

**Table 5:** Inhibition of the growth of phytopathogenic microorganisms by volatile metabolites (VOCs) emitted by *T. atroviride* strain CMU-08 ^1^.

Phytopathogen (Strain)	Inhibition Class in Each Culture Medium	
	VMM	PDA
*B. cinerea* (BC-MICH)	2 (50.25) ^c, d, e^	2 (48.8) ^a, b, c, d, e^
*Fusarium* sp. (FS-MICH)	2 (51.19) ^a, b, c, d, e^	2 (46.42) ^a, b, c, d, e^
*F. mexicanum* (MICMM21)	2 (59.69) ^b, c, d, e^	3 (27.24) ^a, b^
*C. coccodes* (CCJT-1)	2 (59.12) ^c, d, e^	2 (60.83) ^c, d, e^
*C. gloeosporioides* (CG1-MICH)	2 (39.39) ^c, d, e^	2 (46.89) ^a, b, c, d, e^
*C. gloeosporioides* (CG2-MICH)	2 (63.39) ^c, d, e^	1 (68.65) ^c, d, e^
*P. cinnamomi* (PC2-MICH)	1 (70.19) ^c, d, e^	2 (50.39) ^a, b, c, d, e^

^1^ The keys and isolation sites of each phytopathogen are shown in Table 1. Inhibition classes were established according to Sarven et al. [61], class 1 of inhibition toward the phytopathogen being the most efficient for *T*. *atroviride* strain CMU-08 and class 3 the least efficient. All tests were carried out at a temperature of 28 °C. The inhibition percentages in each case are shown in parentheses. Key for the culture media: VMM, Vogel minimal medium; PDA, potato dextrose agar. Different letters represent significant differences (two-way ANOVA with Tukey’s post hoc test, *p* < 0.05). For details, see Section 2.

## Data Availability

All supplementary data are included in the manuscript.

## Supplementary Materials

The following supporting information can be downloaded at: https://www.mdpi.com/article/10.3390/jof10110758/s1, Figure S1: Growth and conidiation of *T. atroviride* strain CMU-08; Figure S2: Growth and conidiation of *T. atroviride* strain CMU-08 at different pH levels; Figure S3: Antagonism assays of *T. atroviride* strain CMU-08 in MEA medium; Figure S4: Inhibition of *B. cinerea* growth by extracellular concentrates of *T. atroviride* strain CMU-08 in a microplate assay; Figure S5: Dose–response analysis of the effect of extracellular lyophilizates of *T. atroviride* strain CMU-08 on the growth of *B. cinerea*; Figure S6: Swiss-Model model and quality parameters of the modeled chitinase encoded by the gen TRIATDRAFT_217415 of *T. atroviride*; Figure S7: Phyre2 quality parameters of the modeled chitinase encoded by the gen TRIATDRAFT_217415 of *T. atroviride*; Figure S8: I-TASSER model and quality parameters of the modeled chitinase encoded by the gen TRIATDRAFT_217415 of *T. atroviride*; Table S1: Category of antagonism and classes of inhibition of the CMU-08 strain of *T. atroviride* against different phytopathogens; Table S2: Selected sequences for phylogenetic and structural analysis of the chitinase encoded in the TRIATDRAFT_217415 locus of *T. atroviride*.
