# Supplementary material for: Polyphasic Characterization of the Biocontrol Potential of a Novel Strain of Trichoderma atroviride Isolated from Central Mexico
Source: J Fungi (Basel). 2024 Nov 1;10(11):758. doi: 10.3390/jof10110758 (PMC11596017; doi:10.3390/jof10110758)
Supplement: Supplementary file 1 [file jof-10-00758-s001.zip › jof-3176182-supplementary.pdf]

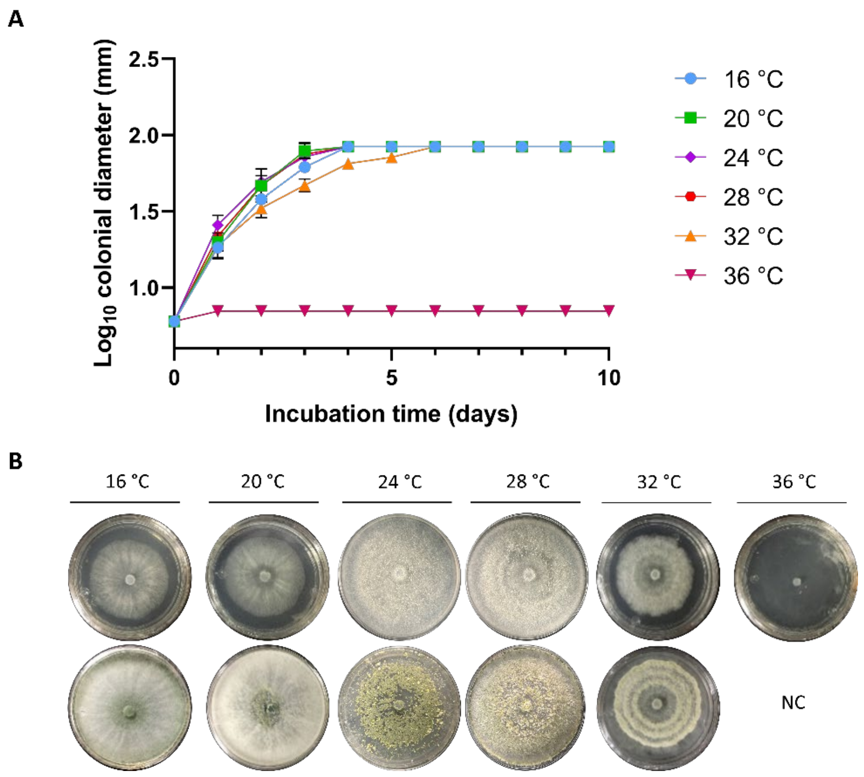

Figure S1. Growth and conidiation of *T. atroviride* strain CMU-08. The strain was inoculated in MEA medium and incubated at the indicated temperatures. Panel A: For growth kinetics, the colonial diameter was measured every 24 hours. The bars represent the standard deviation; those representing the standard deviation are small and thus obscured by the growth kinetics symbols. Panel B: Each culture condition shows the appearance of the colony prior to conidiation (plates on the left at each temperature) and the onset of the conidiation process (plates on the right at each temperature). NC indicates that the strain did not conidiate. The assays were performed in triplicate.

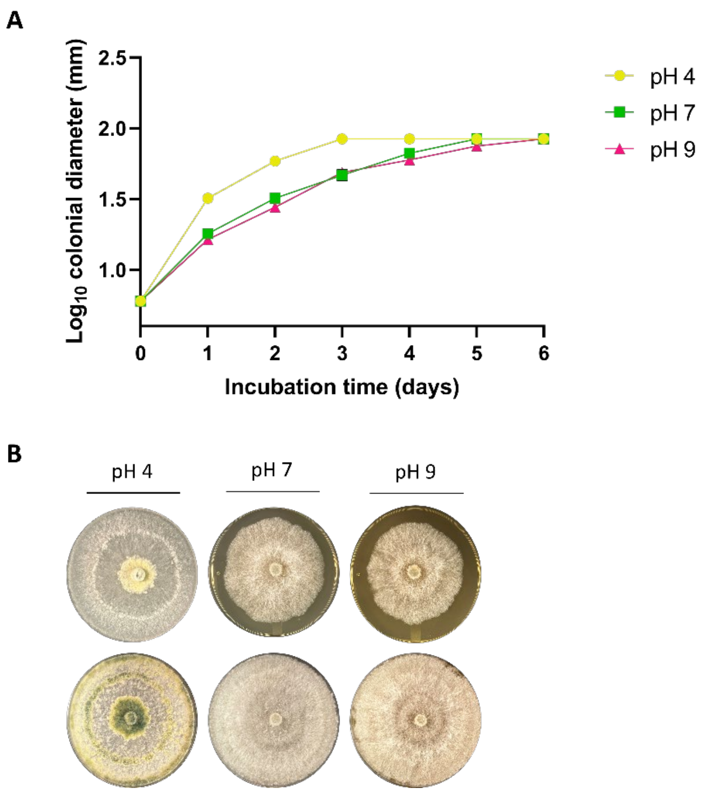

Figure S2. Growth and conidiation of *T. atroviride* strain CMU-08 at different pH levels. Panel A illustrates the growth kinetics on MEA medium, with the colony diameter measured every 24 hours while incubating at 28 °C. Assays were performed in triplicate, and the bars representing standard deviation are small and thus obscured by the growth kinetics symbols. Panel B presents the mycelial development (left column) and the conidiation pattern (right column) of the strain under various growth conditions.

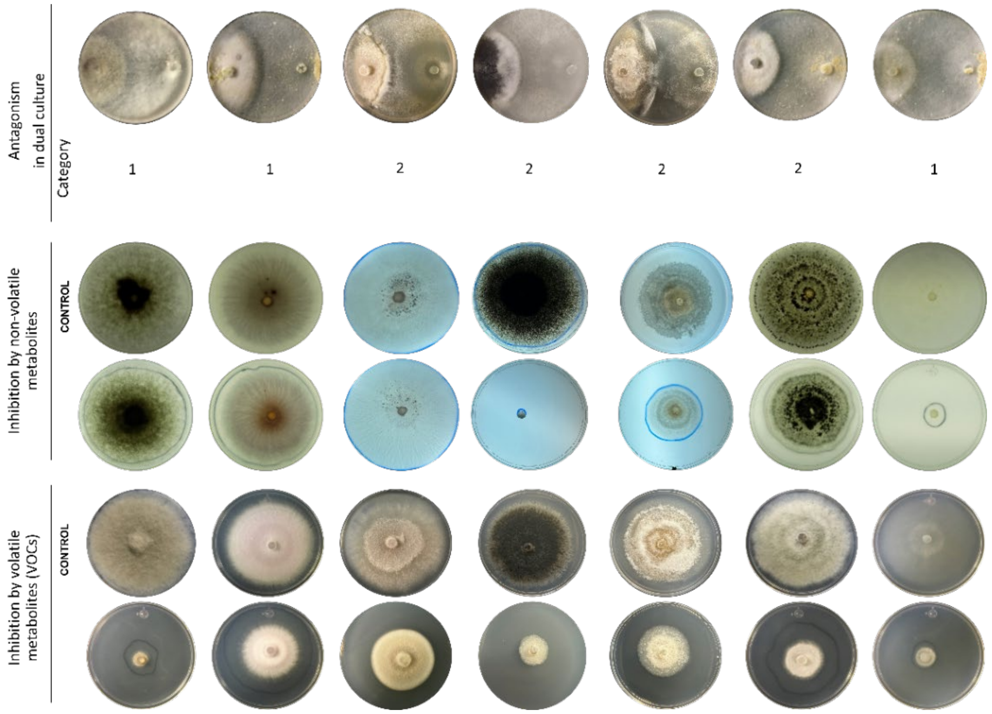

Figure S3. Antagonism assays of *T. atroviride* strain CMU-08 in MEA medium. The image displays confrontation tests in dual culture, as well as inhibition tests using non-volatile and volatile metabolites (VOCs) against different phytopathogens. The column of control plates for each phytopathogen, which do not include *T. atroviride*, is indicated on the left. All tests were conducted at a temperature of 28 °C, and the names of the test phytopathogens are listed at the top. The assays were performed in triplicate

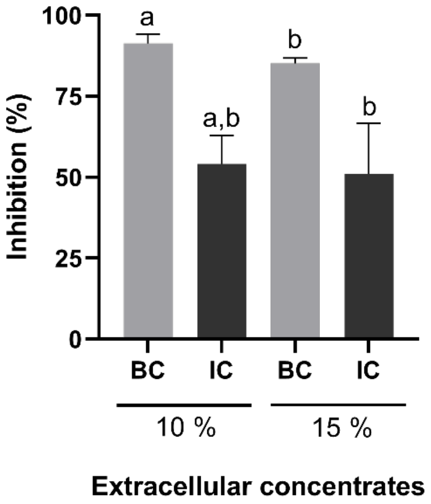

Figure S4. Inhibition of *B. cinerea* growth by extracellular concentrates of *T. atroviride* strain CMU-08 in a microplate assay. The figure illustrates the percentages of *B. cinerea* growth inhibition resulting from the addition of the extracellular medium of strain CMU-08. Key for extracellular media: BC indicates basal condition, and IC indicates induced condition. Three independent assays were performed in triplicate. Bars represent standard error, and different letters denote significant differences (two-way ANOVA with Tukey's *post hoc* test, $P < 0.05$).

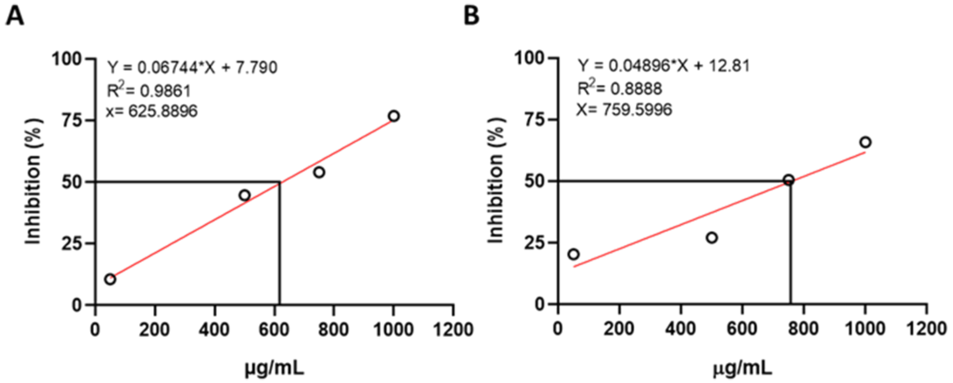

Figure S5. Dose-response analysis of the effect of extracellular lyophilizates of *T. atroviride* strain CMU-08 on the growth of *B. cinerea*. The slope equation and the R2 value are presented for the effect of extracellular lyophilizates under basal condition (BC, panel A) and the induction condition (IC, panel B) of the study strain.

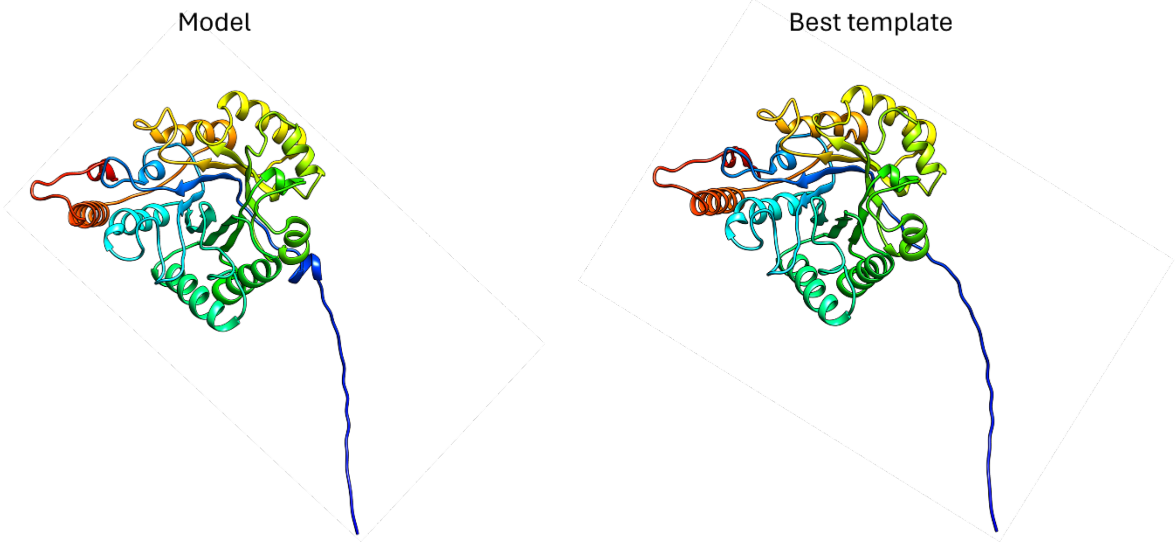

**Biounit Oligo State**	Monomer
**GMQE**	0.89
**QSQE**	-
**Method**	AlphaFold v2
**Seq Similarity**	0.54
**Coverage**	0.99
**Range**	1-361

Trichoderma guizhouense
GH18 endo-N-acetyl-beta-D-glucosaminidase
Gen : A0O28_0064950
Uniprot: A0A1T3CZ84
https://alphafold.ebi.ac.uk/entry/A0A1T3CZ84

Figure S6. Swiss-Model model and quality parameters of the modeled chitinase encoded by the gen TRIATDRAFT_217415 of *T. atroviride.*

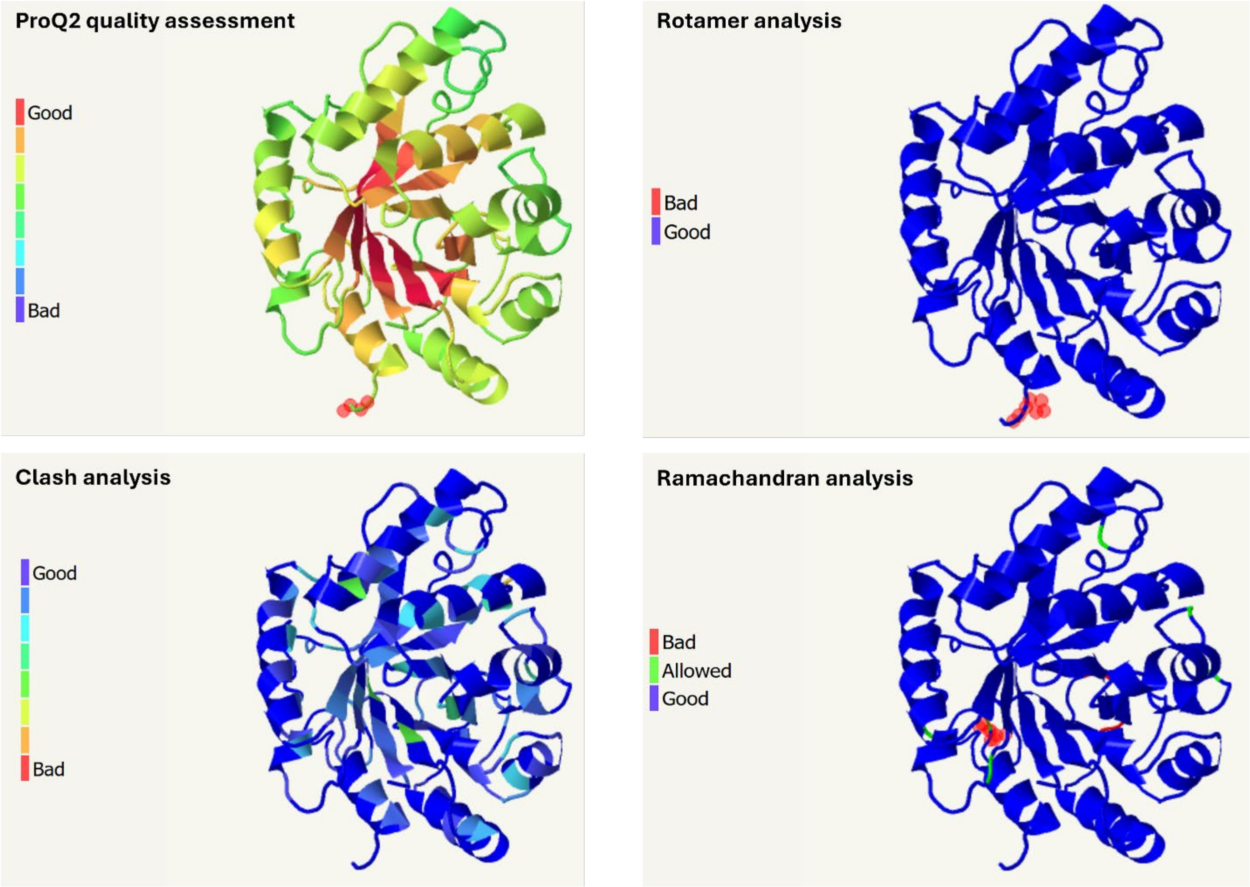

Figure S7. Phyre2 quality parameters of the modeled chitinase encoded by the gen TRIATDRAFT_217415 of *T. atroviride***.** All the parameters were obtained from the model generated as outputs of the Phyre^{2} Investigator option in the Phyre2 server.

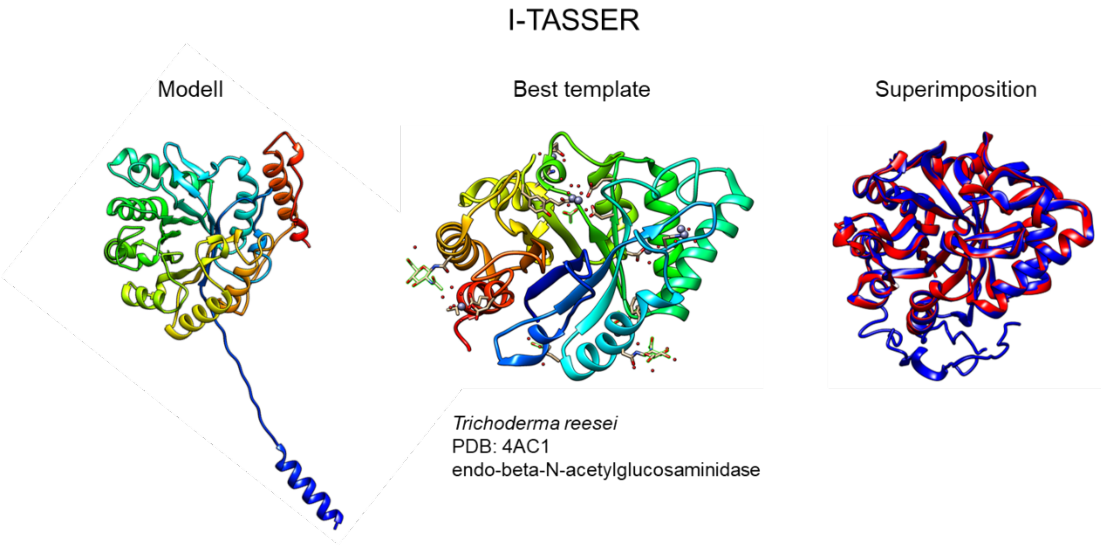

Model parameters			
Iden1	Iden2	Cov	Norm. Z-score
0.77	0.60	0.78	3.55

Figure S8. I-TASSER model and quality parameters of the modeled chitinase encoded by the gen TRIATDRAFT_217415 of *T. atroviride.*

Table S1. Category of antagonism and classes of inhibition of the CMU-08 strain of *T. atroviride* against different phytopathogens[1].

Phytopathogen (strain)	Antagonism level	Inhibition class by non-volatile metabolites	Inhibition class by volatile metabolites (VOCs)
B. cinerea (BC-MICH)	1 ^{a}	2 (38.09) ^{c}	1 (67.06) $^{c, d, e}$
Fusarium sp. (FS-MICH)	1 ^{a}	3 (0) ^{a}	3 (24.6) ^{a}
F. mexicanum (MICMM21)	2 ^{b}	3(0.81) ^{a}	2 (46.84) $^{a, b, c, d, e}$
C. coccodes (CCJT-1)	2 ^{b}	1 (92.68) ^{e}	2 (66.67) $^{c, d, e}$
C. gloeosporioides (CG1-MICH)	2 ^{b}	1 (78.23) ^{d,e}	2 (45.74) $^{a, b, c, d}$
C. gloeosporioides (CG2-MICH)	2 ^{c}	3 (19.44) ^{a,b,c}	2 (39.68) $^{a, b, c}$
P. cinnamomi (PC2-MICH)	1 ^{a}	3 (14.68) ^{a,b}	1 (70.23) $^{c, d, e}$

[1] The keys and isolation sites for each phytopathogen are shown in Table 1. Antagonism levels were established according to Worasatit et al. [59], with level 1 indicating the most efficient antagonism of *T. atroviride* strain CMU-08. Inhibition classes were defined according to Sarven et al. [61], where class 1 represents the most efficient inhibition towards the phytopathogen, and class 3 represents the least efficient. All tests were conducted at a temperature of 28 °C in MEA (malt extract agar). Different letters indicate significant differences (two-way ANOVA with Tukey's post hoc test, $P < 0.05$). For further details, see the Materials and Methods section.

Table S2. Selected sequences for phylogenetic and structural analysis of the chitinase encoded in the TRIATDRAFT_217415 locus of *T. atroviride*.

Microorganism	Specie	Gen/Function	Protein GenBank ID
Fungi	*T. reesei*	*chi18-5*	XP_006968137.1
		chi18-6	DAA05854.1
		chi18-7	EGR45157.1
		chi18-3	XP_006965630.1
		chi18-2	XP_006963889.1
		chi18-11	DAA05859.1
		chi18-4	XP_006965852.1
		chi18-18	DAA05866.1
		chi18-13	XP_006961376.1
		chi18-12	EGR52759.1
		chi18-17	XP_006967940.1
		chi18-14	XP_006969397.1
		chi18-16	XP_006968673.1
		chi18-15	XP_006964800.1
		chi18-8	DAA05856.1
		chi18-10	DAA05858.1
		chi18-9	XP_006968409.1
		chi18-1	EGR52181.1
		chi18-12	DAA05860.1
	T. virens	*ech1*	XP_013960884.1
		ech3	XP_013955725.1
		ech2	XP_013957935.1
		chi18-11	XP_013955716.1
		chi18-18	XP_013951589.1
		chi18-13	ADF57302.1
		cht2 (*chi18-12*)	XP_013958614.1
		cht1.1 (*chi18-17*)	XP_013953573.1
		chi18-14	XP_013961002.1
		chi18-16	XP_013955043.1
		chi18-15	XP_013954420.1
		tvc7	XP_013956203.1
		tvc7	EHK22010.1
		tvc1	XP_013951890.1
		tvc13	XP_013955834.1
		tvc9	XP_013953228.1
		tvc10	XP_013961717.1
		tvc14	XP_013951909.1
		tvc8	XP_013957394.1
		tvc3	XP_013953451.1
		tvc6	XP_013956215.1
		tvc4	XP_013955954.1
		tvc5	XP_013957399.1
		tvc12	XP_013956171.1

	tvc2	EHK23156.1
T. atroviride	*chi18-5*	XP_013943685.1
	chi18-3	XP_013938133.1
	chi18-2	XP_013946881.1
	chi18-11	XP_013939034.1
	chi18-4	XP_013938255.1
	chi18-18	XP_013946026.1
	chi18-13	XP_013942648.1
	chi18-12	XP_013947241.1
	cbm1 (*chi18-17*)	XP_013948974.1
	chi18-14	XP_013949278.1
	chi18-16	XP_013947509.1
	chit36	EHK42260.1
	tac7	XP_013941339.1
	chi18-10	AAZ23945.1
	tac3	XP_013946040.1
	tac6	XP_013947532.1
	tac1	XP_013944966.1
	tac4	XP_013940502.1
	tac5	XP_013941793.1
	tac8	XP_013945500.1
	tac2	XP_013945501.1
	Glycoside hydrolase family 18 protein	XP_013940437.1
	TRIATDRAFT_217415	XM_014089763.1
T. guizhouense	*chi18-13*	OPB46103.1
	chi18-12	OPB42446.1
	chi18-17	OPB41236.1
	chi18-14	OPB40629.1
	chi18-15	OPB37700.1
T. parareesei	*chi18-14*	OTA00337.1
	chi18-15	OTA00434.1
	chi18-8	OTA07931.1
T. arundinaceum	*chi18-17*	RFU72543.1
	chi18-15	RFU80746.1
	chi18-15	RFU80746.1
T. longibrachiatum	*chi18-5*	ACZ63268.1
	Glycoside hydrolase family 18 protein	PTB78921.1
T. tomentosum	*chi18-17*	ADF57314.1
T. viride	*chi18-15*	ADF57289.1
T. polysporum	*chi18-15*	ADF57293.1
T. harzianum	Glycoside hydrolase family 18 protein	XP_024778061.1
T. inhamatum	Chitinase	AAM93196.1

	T. brevicompactum	*chi18-15*	ADF57294.1
	T. ghanense	*chi18-15*	ADF57296.1
	T. asperellum	Glycoside hydrolase family 18 protein	XP_024762386.1
	T. gamsii	Hypothetical protein	XP_018664951.1
	Oidiodendron maius	Glycoside hydrolase family 18 protein	KIM95757.1
Bacteria	*Streptomyces* spp.	Chitinase<br>Chitinase	WP_011028391.1<br>WP_010987038.1
	S. eurocidicus	Chitinase	WP_102919935.1
	S. caatingaensis	Chitinase	WP_049717650.1
	S. mobaraensis	Chitinase	WP_152266141.1
	Spongiactinospora gelatinilytica	Chitinase	WP_111170836.1
	Kribbella albertanoniae	Glycoside hydrolase family 18 protein	WP 132414263.1
	Actinomadura chibensis	Glycoside hydrolase family 18 protein	WP_083980803.1
